# Nanoparticle Targeting Strategies for Lipid and Polymer‐Based Gene Delivery to Immune Cells In Vivo

**DOI:** 10.1002/smsc.202400248

**Published:** 2024-07-30

**Authors:** Manav Jain, Xinjie Yu, Jonathan P. Schneck, Jordan J. Green

**Affiliations:** ^1^ Department of Biomedical Engineering Johns Hopkins University School of Medicine Baltimore MD 21231 USA; ^2^ Institute for NanoBioTechnology, and Translational Tissue Engineering Center Johns Hopkins University School of Medicine Baltimore MD 21231 USA; ^3^ Johns Hopkins Translational ImmunoEngineering Center Johns Hopkins University School of Medicine Baltimore MD 21231 USA; ^4^ Institute for Cell Engineering Johns Hopkins University School of Medicine Baltimore MD 21231 USA; ^5^ Department of Chemical & Biomolecular Engineering Johns Hopkins University Baltimore MD 21218 USA; ^6^ Departments of Pathology and Medicine Johns Hopkins University School of Medicine Baltimore MD 21231 USA; ^7^ Department of Oncology The Sidney Kimmel Comprehensive Cancer Center The Bloomberg∼Kimmel Institute for Cancer Immunotherapy Johns Hopkins University School of Medicine Baltimore MD 21231 USA; ^8^ Departments of Ophthalmology, Neurosurgery, and Materials Science & Engineering Johns Hopkins University Baltimore MD 21218 USA

**Keywords:** biomaterials, biotechnology, gene delivery, immunoengineering, nanotechnology

## Abstract

Lipid nanoparticles and polymeric nanoparticles are promising biomaterial platforms for robust intracellular DNA and mRNA delivery, highlighted by the widespread use of nanoparticle‐ (NP) based mRNA vaccines to help end the COVID‐19 pandemic. Recent research has sought to adapt this nanotechnology to transfect and engineer immune cells in vivo. The immune system is an especially appealing target due to its involvement in many different diseases, and ex vivo‐engineered immune cell therapies like chimeric antigen receptor (CAR) T therapy have already demonstrated remarkable clinical success in certain blood cancers. Although gene delivery can potentially address some of the cost and manufacturing concerns associated with current autologous immune cell therapies, transfecting immune cells in vivo is challenging. Not only is extrahepatic NP delivery to lymphoid organs difficult, but immune cells like T cells have demonstrated particular resistance to transfection. Despite these challenges, the modular nature of NPs allows researchers to examine critical structure–function relationships between a particle's properties and its ability to specifically engineer immune cells in vivo. Herein, several nanomaterial components are outlined, including targeting ligands, nucleic acid cargo, chemical properties, physical properties, and the route of administration to specifically target NPs to immune cells for optimal in vivo transfection.

## Graphical abstract

Delivering targeted nanoparticles (NPs) systemically can enhance immune cell transfection in vivo, with many potential therapeutic applications. There are multiple ways to optimize NP design for targeted nonviral gene delivery to immune cells, including the incorporation of targeting ligands, modifying nanomaterial chemical properties, modifying NP physical properties, and engineering the nucleic acid cargo. Figure made with BioRender.

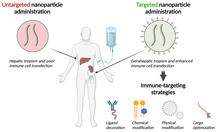

## 1 Introduction

The immune system holds significant therapeutic potential given its ability to localize response to a specific disease site, kill diseased cells in an antigen‐specific manner, minimize off‐target side effects, and retain immunological memory.^[^ ^1^, ^2^ ^]^ Moreover, the rapid emergence of cellular immunotherapies in the past decade highlights how many researchers in academia, industry, and clinic, are utilizing engineered immune cells to treat cancer, autoimmunity, and infectious disease.^[^ ^3^ ^]^ The first chimeric antigen receptor (CAR) T therapy, Kymriah, was approved by the U.S. Food and Drug Administration (FDA) in 2017, with six CAR T therapies approved by the FDA as of May 2024.^[^ ^4^, ^5^ ^]^ These engineered T cell therapies have demonstrated remarkable clinical success against leukemia and are being evaluated in many different disease contexts across hundreds of clinical trials. Beyond T cells, there has been significant work to develop immunotherapies using other cells like macrophages and natural killer (NK) cells for the treatment of different solid and blood‐based tumors.^[^ ^3^, ^6^ ^]^

Despite the promise of these different immune cell therapies, they all suffer from significant processing and manufacturing concerns that prevent widespread adoption. Namely, to prevent rejection, these therapies are autologous, meaning that the patient's own immune cells must be harvested, engineered ex vivo, expanded until a therapeutic dose is reached, and then reinfused back into the patient.^[^ ^7^ ^]^ This process is incredibly expensive, with a single CAR T infusion costing a patient hundreds of thousands of dollars.^[^ ^8^ ^]^ Additionally, there are several points during the manufacturing process where issues can occur, including the risk of cell contamination, poor cell expansion, or cell quality loss during transport from the leukapheresis facility to the processing facility.^[^ ^9^ ^]^ Furthermore, ex vivo CAR T generation/expansion can be slow, ranging from 7 to 12 days.^[^ ^10^ ^]^ This can be potentially threatening to the patient, as they may not be able to tolerate this long waiting period. Ultimately, these issues associated with ex vivo immune cell manufacturing render these therapies inaccessible to most patients. Although work is being performed to improve this ex vivo processing through the development of allogeneic cell therapies and novel bioreactors for faster cell expansion, these approaches may still face challenges regarding cost, manufacturing complexity, and limited efficacy against most solid tumors.

Gene delivery is a promising alternative therapy. DNA and mRNA nanoparticles (NPs) have been shown to transfect human cells in vivo, highlighted by the lipid nanoparticle (LNP) mRNA vaccines used during the COVID‐19 pandemic.^[^ ^11^ ^]^ This same nucleic acid nanotechnology can be implemented in the context of immune cell therapy to potentially transfect and engineer immune cells in vivo, thereby serving as a cell‐free, off‐the‐shelf therapeutic for patients, rather than the current autologous, patient‐specific adoptive cell therapies.^[^ ^12^ ^]^

Given this immense promise, there is a great need for generating novel vectors capable of robust immune cell transfection in vivo. Currently, the primary vectors used for in vivo gene delivery are LNPs, polymeric nanoparticles (PNPs), and viruses.^[^ ^13^ ^]^ Several different viral vectors including adeno‐associated and retroviral vectors have been used in both ex vivo and in vivo gene editing contexts. While these approaches are a viable option for immune cell engineering, they involve several key drawbacks regarding the fact that viral vectors are costly to produce, have a limited nucleic acid packaging capacity (5–10 kb), can have random, unwanted integration into the host genome, and are immunogenic, meaning that they can induce a harmful host immune response in vivo.^[^ ^14^, ^15^, ^16^ ^]^ While nonviral gene delivery vectors like LNPs and PNPs tend to demonstrate lower gene transfer efficiency than viral vectors, nanoparticles (NPs) have a much higher cargo capacity and reduced safety risks in vivo.^[^ ^11^ ^]^ Most importantly, these NP‐based approaches are highly modular, meaning that different chemical and physical parameters of the nanomaterial can be easily varied to optimize in vivo transfection of the desired immune cell type.

Among nonviral nucleic acid vectors, LNPs are the most established platform given their FDA approval as a COVID‐19 vaccine and ongoing investigation in many other disease areas.^[^ ^17^, ^18^ ^]^ LNPs are traditionally composed of four lipids: an ionizable cationic lipid, a helper phospholipid, a helper cholesterol lipid, and a poly‐ethylene glycol (PEG)‐lipid.^[^ ^19^, ^20^ ^]^ The ionizable lipid plays the most crucial role in LNP intracellular delivery, as the positive charge enables nucleic acid encapsulation and the tertiary amines in the lipid determine LNP pKa and enable endosomal escape.^[^ ^21^ ^]^ In addition to lipids, there are also many different types of polymers used to formulate NPs for gene delivery, including poly(β‐amino ester)s (PBAE), polyethylenimine (PEI), polyamidoamine (PAMAM), and poly(amino*‐*co‐ester)s (PACE).^[^ ^22^ ^]^ Like the ionizable lipid, these polymers are generally cationic to self‐assemble with the negatively charged nucleic acid to form a NP. Additionally, these polymers often contain primary, secondary, and tertiary amines that assist in NP uptake and endosomal escape.^[^ ^23^ ^]^ Among PNPs, PEI has traditionally been the most commonly used polymer. However, PEI is also observed to cause significant cytotoxicity, and other polymers, such as PBAEs are being widely adopted.^[^ ^24^ ^]^ Like the different components in LNPs, these polymers are highly modular, meaning that many different polymer structures can be easily synthesized.

**Figure 1:**
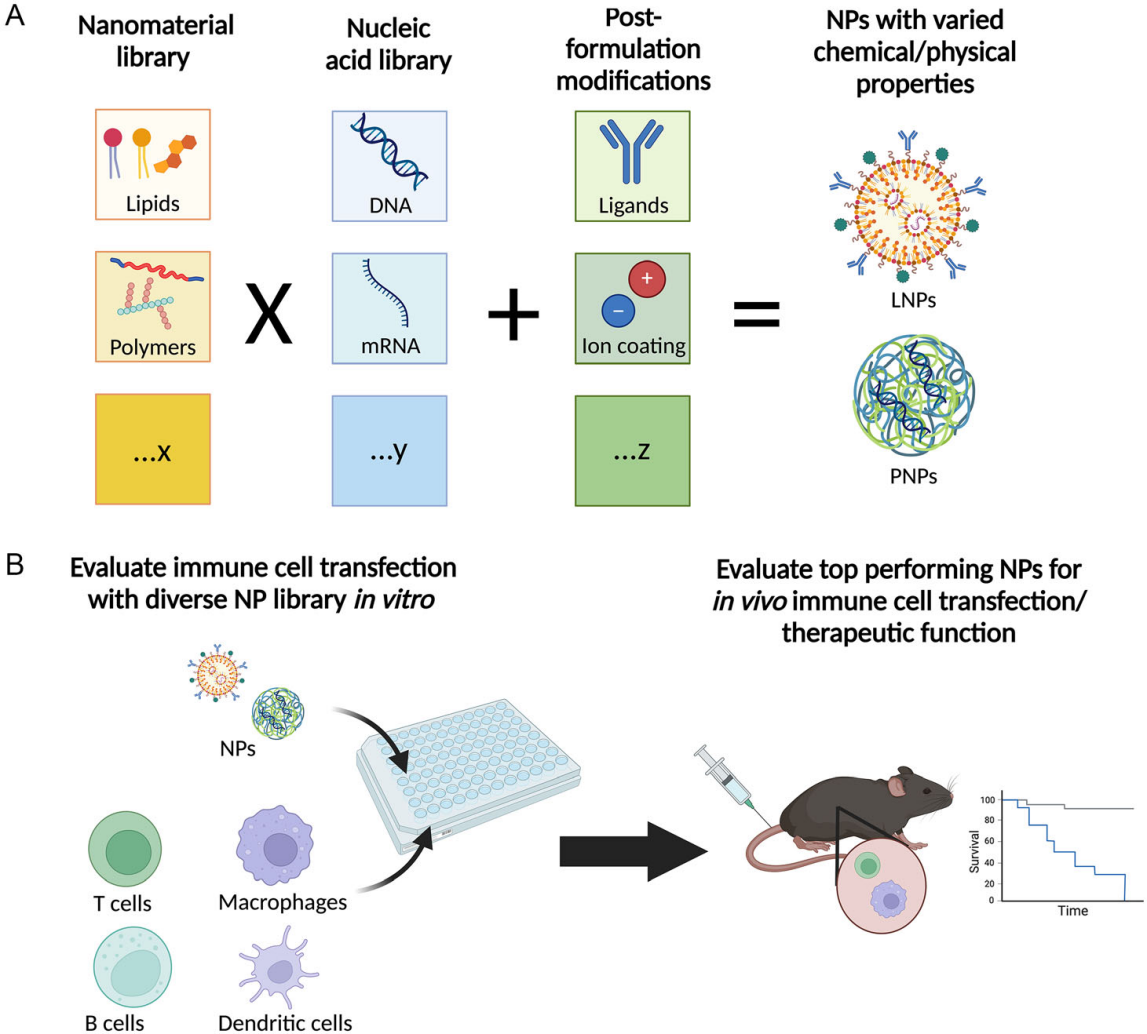
NP synthesis and screening is a modular process. A) Different nanomaterial building blocks, including many kinds of lipids (for LNPs) and polymers (for PNPs) can self‐assemble with many nucleic acid cargos encoding different therapeutic constructs to create NPs with diverse chemical and physical properties. These NPs can be further engineered through surface modification or ligand conjugation. This combinatorial synthesis process gives rise to a library of NPs with diverse structural features. B) These diverse NP libraries can be screened in vitro to identify top‐performing formulations and characterize NP structure–function relationships. Finally, top‐performing formulations identified from in vitro screening can be delivered in vivo to evaluate their ability to transfect endogenous immune cells and engineer therapeutic function. Figure made with BioRender.

This potential chemical diversity highlights the importance of thinking about in vivo immune cell transfection from a biomaterials‐based approach. As researchers continue to design different types of LNP and PNP formulations, key structure–function relationships between different NP characteristics and their efficacy in vivo have begun to emerge. In this review, it is examined how the different components of a gene delivery NP can be engineered and optimized to transfect specific immune cells in vivo. As outlined in **Figure** **1**, nanomaterial building blocks and cargo are combined to form a NP which can then undergo post‐formulation modifications. Given that there are many different components that can be used in each step of this modular fabrication process, the potential chemical diversity of NPs that can be used to target immune cells is very large.

In this review, four critical parameters of NPs are outlined that drive interactions between the particle and an immune cell: the presence of a targeting ligand on the NP surface, NP material chemical properties, NP physical properties, and properties of the nucleic acid cargo inside of the NP. The key structure–function relationships that help target NPs to immune cells in vivo are outlined along with a discussion of delivery route‐mediated targeting and the role of assistive biomaterials to enhance transfection.

Given the transformative potential of using NPs for in vivo immune cell therapy, there have already been several excellent reviews published on this topic since 2020 alone^[^ ^13^, ^25^, ^26^, ^27^, ^28^, ^29^, ^30^, ^31^ ^]^ focused on the role of active, or ligand‐mediated, targeting of NPs to immune cells. This is understandable, as active immune cell targeting has currently been shown to be a very effective strategy and is being explored commercially by different companies such as Capstan Therapeutics and Umoja Biopharma.^[^ ^32^, ^33^ ^]^ While this more conventional approach of ligand‐based targeting is explored in this review, the other ways that NPs can be engineered to optimize immune cell targeting are also well‐described. By approaching the challenge of immune cell transfection from more of a biomaterials‐based perspective, recent findings regarding structure–function relationships can be utilized to better inform future NP design. A summary of the five major NP targeting strategies and example applications of each approach that will be discussed in this review can be seen in **Table** **1**.

**Table 1:** Summary of five major targeting approaches for enhancing immune cell transfection in vivo: ligand‐, chemical‐, physical‐, cargo‐mediated, and delivery route‐mediated targeting. In addition to outlining the central approach behind each targeting strategy, an assessment of how well‐characterized each strategy is in literature for specifically transfecting immune cells in vivo. Additionally, two relevant examples that are discussed in the review are provided for each targeting strategy to outline the specific nanomaterial, nucleic acid cargo, cell‐type targeted, and novel targeting mechanism employed in the study.^[^ ^50^, ^63^, ^91^, ^92^, ^128^, ^148^, ^173^, ^181^, ^189^, ^196^ ^]^

Targeting strategy	Central approach	Approach established for in vivo immune cell transfection?	Example nanomaterial/cargo	Specific targeting mechanism	Immune cell targeted
Ligand‐mediated targeting	Conjugating ligands to NP surfaces that are specific to receptors or proteins expressed on immune cells can enhance transfection.	+ + + + +	LNP/single variable domain heavy chain (VHH) antibody mRNA^[^ ^50^ ^]^	Conjugation of pMHC I molecule improves NP binding with cognate cell	Virus‐specific CD8 + T cells
			PNP (PBAE)/IRF5 mRNA^[^ ^63^ ^]^	Conjugation of dimannose to NP improves binding with CD206 on specific cells	Tumor‐associated macrophages
Chemical targeting	Modifying the chemical properties of nanomaterial building blocks or incorporating other chemicals can enhance extrahepatic delivery and improve uptake, endosomal escape, and transfection specifically in immune cells.	+ + +	LNP/CAR mRNA^[^ ^91^ ^]^	Incorporation of anionic 18:1 PA lipid improves β2‐GPI adsorption and spleen tropism	Splenic T cells
			PNP (PBAE)/OVA mRNA^[^ ^92^ ^]^	Incorporation of lipophilic content in polymer backbone improves uptake and transfection	Splenic dendritic cells
Physical targeting	Modifying the physical properties of the nanomaterial can promote favorable interactions between a particle and an immune cell, leading to enhanced uptake, biodistribution, and modulation of specific immune cells.	+	Silica NPs/no cargo^[^ ^128^ ^]^	Modifying NP size >300 nm increases uptake via phagocytic pathways	Macrophages
			PEG microparticle/no cargo^[^ ^148^ ^]^	Softer MPs (50 kPa) demonstrate higher T cell binding, and avoid macrophage uptake than stiffer ones (5000 kPa)	T cells
Cargo‐mediated targeting	Modifying the nucleic acid cargo in a particle can enhance the translation of that gene in specific immune cells, even if the particle is taken up by nontarget cells.	+ +	PNP (PEG‐PLGA)/Cas9 + sgRNA pDNA with CD68 promoter^[^ ^173^ ^]^	CD68 plasmid promoter only expressed in CD68+ cells	Macrophages, monocytes
			LNP/CAR IVT mRNA^[^ ^181^ ^]^	m1ψ ‐ modified mRNA demonstrates higher transfection	Macrophages, T cells
Delivery route‐mediated targeting	Codelivery of particles with other biomaterials can enhance controlled release, immune cell recruitment, and transfection. Additionally, altering the site where particles are injected can enhance extrahepatic delivery	+ +	PNP (mPEG‐PCL‐PEI)/CAR encoding plasmid + supra‐molecular hydrogel^[^ ^196^ ^]^	Injection of a NP‐hydrogel composite proximal to the tumor reprogrammed nearby immune cells	T cells
			PNP (alginate)/miRNA‐223 + adhesive hydrogel^[^ ^189^ ^]^	Injection of inflammatory hydrogel recruited immune cells which were then transfected	Macrophages

## 2 Ligand‐Mediated Targeting

### 2.1 Motivation for Active Targeting

As previously mentioned, there are many barriers to extrahepatic delivery of NPs, which makes in vivo gene delivery to immune cells that reside in lymphoid organs like the spleen and lymph node very challenging. Upon intravenous injection, the serum protein ApoE often adsorbs to the surface of LNPs, thereby directing these particles to the liver.^[^ ^34^ ^]^ Additionally, many PNP complexes show poor stability in serum, which in turn hinders their efficacy.^[^ ^35^ ^]^ To mitigate these endogenous barriers to passive immune cell targeting, a variety of NP engineering approaches have been employed to functionalize these biomaterials with different targeting ligands specific to a variety of immune cells, including antibodies, different types of sugars, and other macromolecules that interact with receptors on specific immune cells. Here, these broad classes of active targeting ligands are discussed. Additionally, the major challenges associated with ligand‐mediated targeting are outlined, highlighting the need for alternative engineering strategies to address these shortcomings.

### 2.2 Approaches

#### 2.2.1 Antibodies

Many different types of antibodies and antibody fragments have been utilized for targeted NP drug delivery.^[^ ^36^ ^]^ In the context of gene delivery to immune cells, antibody‐functionalized NP engineering approaches have been used to generate CAR T cells in situ. Seminal work to design antibody‐NPs for active T cell targeting has been pioneered in the lab of Mattias Stephan at the Fred Hutchinson Cancer Center. In the earliest iteration of his group's work on in situ CAR‐T cell generation, Smith et al. designed a polymeric PBAE NP with anti‐CD3 F(Ab’)2 fragments conjugated to the particle surface.^[^ ^37^ ^]^ Upon intravenous administration, these PNPs were able to transfect circulating T cells with DNA encoding for an anti‐CD19 CAR construct and generate CAR T cells in situ. The authors found that the CD3‐targeted NPs were capable of binding and transfecting T cells with more specificity than untargeted particles in vivo. This translated to therapeutic efficacy, as mice that received the CD3‐targeted NPs enabled leukemia regression with the same efficacy as adoptively transferred, ex vivo virally transduced T cells. Ultimately, this study highlights the promise of using gene delivery NP technology to reprogram endogenous T cells and bypass much of the costly, complicated ex vivo engineering associated with CAR‐T manufacturing.

Despite the exciting anticancer responses demonstrated by Smith et al. the therapeutic efficacy of this system was predicated on the integration of CAR DNA from the NP into the host T cell genome through the use of a transposase.^[^ ^37^ ^]^ While effective, this form of DNA delivery carries drawbacks due to the potential for unwanted, potentially harmful genomic rearrangement due to the random, permanent insertion of the DNA into the host cell genome via the transposase.^[^ ^38^, ^39^ ^]^ To address these concerns, a study by Parayath et al. advanced the research by Smith et al. by designing PBAE NPs that were functionalized with anti‐CD8 antibodies and delivered in vitro‐transcribed mRNA encoding for a therapeutic CAR or T cell receptor (TCR).^[^ ^40^ ^]^ These NPs were also capable of robust CAR mRNA delivery to T cells in vivo and inducing anticancer responses in mice.

These two studies from the Stephan group in 2017 and 2020 highlight early reported instances of success at using antibody‐decorated NPs for targeted DNA and mRNA delivery to T cells in vivo, respectively. Since then, there has been significant innovation in this space to optimize the biomaterial, targeting moiety, and overall NP synthesis process for immune cell reprogramming in a variety of therapeutic contexts. A novel application of this technology was demonstrated by Rurik et al. where the researchers developed ionizable LNPs coated with antibodies targeting CD5, a scavenger receptor expressed on T cells and some B cells.^[^ ^41^, ^42^ ^]^ The CD5‐targeted LNPs delivered mRNA to T cells encoding for a CAR against fibroblast activation protein (FAP), a protein that is upregulated during cardiac fibrosis and can lead to decreased heart function.^[^ ^43^ ^]^ Building on previous work with ex vivo‐generated, FAP‐specific CAR T cells, the researchers demonstrated that the targeted LNPs efficiently transfect splenic T cells in both reporter luciferase/Ai6 models and in a therapeutic FAP‐CAR model while the nontargeted LNPs did not demonstrate high splenic transfection. The in vivo‐generated CAR T cells were then able to eliminate fibrosis in the heart and restore cardiac function, demonstrating the potential of targeted mRNA NPs to reprogram immune cells for use in many therapeutic contexts beyond cancer. Moreover, the transient nature of mRNA expression makes diseases like cardiac fibrosis potentially better suited for treatment with in vivo CAR T gene therapy because these diseases do not persist for the same duration as cancer and the eventual loss of CAR construct expression could help mitigate long‐term side effects.

There has also been significant research into optimizing the form of the antibody conjugated to NP surface. Several groups have investigated the effect of conjugating F(Ab’)2, F(Ab’), single chain variable fragments (scFv), and peptide‐MHC monomers (pMHC) to NPs for immune cell transfection in vivo.^[^ ^44^ ^]^ A study by Billingsley et al. generated Ab‐LNPs that consisted of a LNP conjugated to either an anti‐CD3, anti‐CD5, or anti‐CD7 F(Ab’).^[^ ^45^ ^]^ After optimizing the antibody density on the surface of the particle, the researchers showed that NPs coated with anti‐CD3 were capable of transfecting endogenous T cells with GFP mRNA, with GFP expression peaking at 6 h in the blood and at 24 h in the spleen. Interestingly, NPs functionalized with anti‐CD5 demonstrated poor in vivo transfection of T cells in the blood and spleen, whereas the CD5‐targeted NPs used in the previously mentioned study by Rurik et al. demonstrated high efficacy. These discrepancies in in vivo transfection efficiency can most likely be attributed to LNP chemical formulation, dosage, and readout time. Additionally, a recent study by Metzloff et al. outlined the development of LNPs coated with anti‐CD3 and anti‐CD28 F(Ab’) fragments.^[^ ^46^ ^]^ The researchers demonstrated that NPs with both anti‐CD3 and anti‐CD28 on their surface transfected naïve T cells significantly more than LNPs with only anti‐CD3 or anti‐CD28. Although this study primarily focuses on ex vivo transfection of T cells, the high CAR expression achieved by the researchers (>80%) with particles that had both anti‐CD3 or anti‐CD28 F(Ab’) fragments demonstrate the potential of using multiple targeting moieties on the same particle. Moreover, there has been research demonstrating that T cell activation, driven by signaling through the TCR/CD3 and costimulation via CD28, improves NP uptake.^[^ ^47^ ^]^ As such, NPs that incorporate both TCR and costimulation targeting moieties on their surface could potentially demonstrate higher T cell transfection in vivo as well. The use of F(Ab’) fragments instead of whole monoclonal antibodies also potentially provides several benefits: they retain the T cell targeting and binding ability of whole antibodies, and they are smaller, meaning the overall size of the NP does not significantly change after conjugation, and they do not have an Fc region, which could be inflammatory or be result in unwanted uptake by macrophages or other cells that possess Fcγ receptors.^[^ ^48^ ^]^

**Figure 2:**
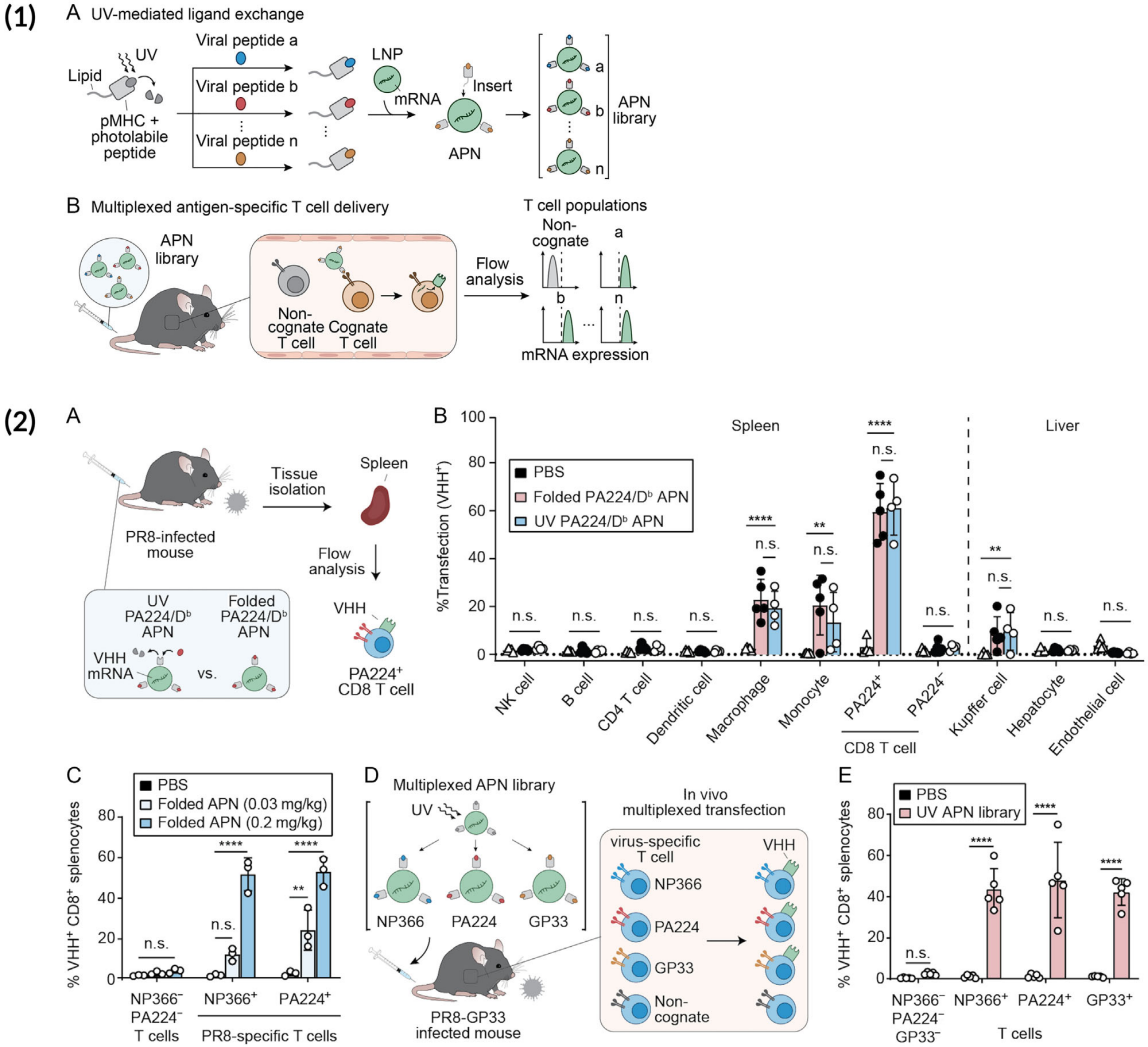
1) (A) Overview of antigen‐presenting NP (APN) synthesis process whereby pMHC monomers are loaded via UV‐mediated peptide folding and conjugated to LNP surface and (B) can then be delivered to mice to assess antigen‐specific transfection. 2) (A) APNs were synthesized using either traditional peptide folding or UV‐mediated peptide exchange and (B) delivered to PR8‐infected mice, (C) where they demonstrated robust antigen‐specific T cell transfection and minimal off‐target T cell transfection. (D) A multiplexed library of APNs specific to the top three epitopes associated with PR8 was then formed using UV peptide exchange and delivered to mice, (E) where it demonstrated robust transfection of each of the three antigen‐specific populations. Reproduced (Adapted) with permission.^[^ ^50^ ^]^ Copyright 2022, Science Advances.

While many of the T cell targeting approaches utilize NPs decorated with targeting moieties like CD3, CD5, and CD8, there is also significant merit to transfecting specific T cell populations that are responsive to a certain antigen while leaving other T cell populations untouched and avoiding overstimulation of the host immune system.^[^ ^49^ ^]^ In a study by Su et al. the researchers utilized LNPs conjugated to class I pMHC monomers to transfect antigen‐specific CD8+ cells.^[^ ^50^ ^]^ These LNPs were synthesized with a UV light‐mediated pMHC loading method that allowed for simple formulation of targeted mRNA LNPs that are specific to a certain TCR. The researchers found that pMHC LNPs bound and efficiently transfected their cognate T across several different transgenic mouse models/alleles (P14, OT‐I, PMEL). Additionally, the researchers developed a multiplexed library of three different pMHC LNPs that were specific to three influenza epitopes. After delivering this multiplexed LNP library to a mouse infected with influenza, the researchers saw robust transfection in the three dominant antigen‐specific T cell populations associated with the flu. The overall workflow of the approach is outlined in **Figure** **2**. This work shows the potential of using other targeting moieties beyond broad pan‐T antibodies to specifically engineer antigen‐specific T cells.

Although the majority of antibody‐mediated NP targeting has been focused on engineering T cells, there has also been significant research to leverage similar approaches for transfecting other immune cells, such as macrophages. Macrophages also represent a therapeutically appealing cell type due to their role in many different diseases, different phagocytosis mechanisms, and the potential to exhibit both proinflammatory and anti‐inflammatory effects.^[^ ^51^ ^]^ A study by Veiga et al. developed LNPs conjugated to an anti‐Ly6c antibody to target and reprogram inflammatory leukocytes.^[^ ^52^ ^]^ By transfecting leukocytes with LNPs carrying IL10‐encoding mRNA, these leukocytes then produced anti‐inflammatory cytokines which helped relieve the burden of inflammatory bowel disease. Additionally, a study by Parayath et al. employed PBAE mRNA NPs functionalized with an anti‐CD64 F(Ab’) to specifically target the NPs to macrophages.^[^ ^40^ ^]^ CD64, or the Fcγ receptor‐1, is normally expressed on the surface of cells like macrophages and is overexpressed on monocytes, macrophages, and neutrophils in the context of many autoimmune diseases.^[^ ^53^ ^]^ By utilizing NPs that had targeting ligands specific to the innate immune cells that were driving an autoimmune response in lupus nephritis, the researchers were able to specifically transfect these cell types with mRNA that downregulated key inflammatory signatures, thereby reducing the burden of the disease. Ultimately, these studies highlight the potential of using antibody‐mediated targeting to engineer innate immune cells for anti‐inflammatory applications. Moreover, given the recent clinical interest in CAR macrophages and the inherent phagocytic and inflammatory potential of macrophages, there is also potential to utilize similar antibody‐mediated macrophage targeting approaches to engineer cytotoxic responses.^[^ ^54^ ^]^

Although there have been fewer published studies using targeted NPs to target other immune cells like NK cells, B cells, and neutrophils, there is still great potential to apply many of the concepts that guided the development of T cell‐targeting NPs to transfect these other cell types. Most immune cells have specific cell surface markers that are heavily conserved and have minimal overlap with other cell types. For example, NK cells express surface markers like CD56 and CD16 at significantly higher levels than other immune cells.^[^ ^55^, ^56^, ^57^ ^]^ Additionally, CD19 and CD20 are surface markers canonically associated with B cells. Furthermore, there are many different surface markers associated with different lineages and M1/M2 subtypes of macrophages, suggesting that different targeting antibodies may result in improved delivery to different macrophage subsets.^[^ ^58^, ^59^, ^60^ ^]^ Many of the T cell targeting NP platforms outlined above are highly modular, meaning that the ligand employed in the study could be substituted for a different antibody to potentially transfect a different cell type.^[^ ^61^ ^]^

#### 2.2.2 Carbohydrates

Sugars are an appealing ligand for NP delivery to certain immune cells that have been reported to exhibit carbohydrate‐dependent uptake. For example, macrophages possess a mannose receptor, CD206, that mediates phagocytosis.^[^ ^62^ ^]^ This mannose‐driven phagocytosis can be harnessed for NP uptake and efficient gene delivery. In a study by Zhang et al. the researchers designed PBAE NPs coated with dimannose as an active targeting ligand.^[^ ^63^ ^]^ NPs coated with the dimannose were able to effectively transfect immunosuppressive tumor‐associated macrophages (TAM) in vivo with mRNA encoding for interferon regulatory factor 5 (IRF5) and its associated kinase (IKKβ). Expression of this IRF5 construct led to macrophages being polarized away from the immunosuppressive/TAM phenotype and toward an inflammatory/M1 phenotype that reduced tumor burden in several solid tumors. In addition to macrophage repolarization, a similar mannose‐based targeting approach was used to generate CAR macrophages in vivo. Kang et al. developed PNPs consisting of PEI conjugated to mannose for delivery of a DNA construct that encodes a CAR and interferon‐γ.^[^ ^64^ ^]^ Upon transfection with the mannose‐targeted NPs, the production of interferon‐γ polarized macrophages toward an inflammatory M1 phenotype and the expression of the CAR construct enabled these M1 macrophages to elicit antitumor responses.

In addition to the mannose‐targeted PNPs used for macrophage engineering mentioned above, mannose conjugation to the surface of LNPs has been used to improve their delivery to antigen‐presenting cells (APCs). Goswami et al. demonstrated that an LNP delivering antigen‐encoding nucleic acid and conjugated to a mannose‐cholesterol amine improved uptake by APCs in vivo and led to rapid antibody titers against the vaccine antigen.^[^ ^65^ ^]^ Moreover, these mannosylated LNPs were able to elicit rapid and robust antibody responses through both intramuscular and intradermal administration. In another similar study, researchers functionalized mRNA liposomes with mannans of varying repeat lengths that were known to associate with the DC‐SIGN receptor expressed on APCs.^[^ ^66^ ^]^ Interestingly, liposomes with mannans that were at least 2 monosaccharides in length were necessary for robust APC transfection in vivo and strong antigen‐specific vaccine responses.

To date, mannose has been the most extensively explored sugar for use in targeted gene delivery to immune cells, largely due to the expression of CD206 on the surface of many macrophages and APCs. However, these monocytes also possess several other carbohydrate receptors. For example, macrophages possess β‐glucan receptors that enable phagocytosis of many plant, fungi, and bacteria membranes.^[^ ^67^, ^68^ ^]^ This β‐glucan phagocytosis mechanism was utilized by Ren et al. who developed yeast cell wall‐coated poly(lactic*‐*co‐glycolic acid) (PLGA) NPs for enhanced endocytosis and drug delivery to macrophages.^[^ ^68^ ^]^ Additionally, macrophages possess a folate receptor that is expressed at higher levels when the macrophage is in an activated, inflammatory state.^[^ ^69^ ^]^ As such, Poh et al. developed a folate‐targeted polymeric dendrimer NP that would specifically accumulate at sites of macrophage‐mediated inflammation in a mouse model of atherosclerosis.^[^ ^70^ ^]^ Although these studies are not focused on gene delivery applications, they demonstrate viable methods of designing different sugar‐coated NPs to improve delivery to immune cells like macrophages. Moving forward, additional research into the efficacy of nucleic acid NPs functionalized with sugars like β‐glucan and folate in vivo would be fruitful to further advance gene therapy targeting strategies.

#### 2.2.3 Other Macromolecules

While antibodies and carbohydrates make up most of the ligands used in active NP targeting approaches, there are other macromolecules that have been shown to redirect NPs toward certain immune cells in vivo. These approaches are highly varied across the type of targeting moiety employed and the cell type of interest that is targeted.

Different peptides have been utilized to modulate NP tropism and immune cell delivery. In a study by Jain et al. the researchers developed alginate NPs that encapsulated IL‐10 DNA and were functionalized with tuftsin, a peptide that specifically binds with macrophages and neutrophils and promotes phagocytosis.^[^ ^71^, ^72^, ^73^ ^]^ The alginate‐tuftsin NPs were used in a therapeutic model of arthritis, whereby the targeted NPs accumulated at the site of inflammation. By transfecting the inflammatory macrophages with IL‐10 DNA, the NPs polarized the arthritic environment toward being immunosuppressive, thereby reducing inflammation. Although this approach used a novel peptide to enhance DNA delivery to macrophages, the overall approach is similar to those used in carbohydrate‐based targeting: macrophages and other monocytes have macromolecule‐specific receptors that enhance uptake and phagocytosis of NPs.

Specific peptide sequences can be employed in NP design to target the particle to the immune cell of interest. Li et al. designed a polymeric DNA NP whereby the polymer was an amphiphilic peptide‐SA monomer consisting of stearic acid conjugated to a nuclear localization peptide sequence and a macrophage‐targeting peptide sequence.^[^ ^74^ ^]^ This novel PNP was able to generate CAR‐macrophages in vivo that were specific to *Staphylococcus aureus* and able to prevent infection. A similar, peptide‐based targeting approach was utilized by Cruz et al. for targeted drug delivery to neutrophils.^[^ ^75^ ^]^ In the study, the researchers designed polymeric NPs that had a neutrophil elastase binding protein on the end of the monomer, allowing the NP to bind to neutrophil elastase, a protein expressed by activated neutrophils. When drug loaded, neutrophil‐targeting NPs were delivered intravenously, they selectively reduced neutrophil activity and thrombosis. Although this study used peptide‐functionalized NP for drug delivery, a similar approach could be employed for gene delivery to neutrophils, a cell type that has historically not been studied in gene therapy contexts.

Additionally, albumin is a serum protein that has frequently been leveraged in different drug delivery contexts given its ability to enhance circulation time and extrahepatic delivery. For example, Azevedo et al. designed PLGA‐PEG NPs encapsulating insulin and conjugated to human albumin. These albumin‐functionalized PNPs demonstrated enhanced binding to human neonatal Fc receptor (FcRn) in vitro and reduced glycemia burden when orally administered in a mouse model of diabetes.^[^ ^76^ ^]^ Neonatal Fc receptors play an important role in mediated transport across mucosal barriers, and as such, the incorporation of albumin as a targeting ligand could help improve the efficacy of oral and inhaled NP delivery.^[^ ^77^ ^]^ Moreover, this concept of albumin‐mediated targeting has been explored through the targeting of endogenous albumin. Several studies have outlined the design of modified siRNA, peptide vaccines, and extracellular vesicles that bind serum albumin when administered in vivo to improve circulation time to promote drug delivery to the tumor, lung, or lymph node, respectively.^[^ ^78^, ^79^, ^80^ ^]^ Many of these findings regarding the importance of albumin binding in improving drug delivery efficacy can be easily translated to PNP and LNP design. In addition to being physically conjugated to the NP, albumin can also be coformulated with a nanomaterial to form an albumin‐based DNA or RNA NP for gene delivery.^[^ ^81^ ^]^ These albumin‐based NPs can employ Fc receptor‐mediated trafficking and enhanced circulation to transfect the desired organ or cell type in a manner similar to the studies outlined above.

### 2.3 Limitations of Ligand‐Mediated Targeting

Although active targeting has been shown to be an effective method for redirecting NPs toward specific immune cell types, there are several limitations associated with this approach. Many of these potential drawbacks are associated with antibody‐mediated targeting approaches, as the conjugation of monoclonal antibodies specific to immune cell surface markers can elicit unwanted immune responses. For example, in the previously outlined study by Billingsley et al. the researchers discovered that LNPs functionalized with anti‐CD3 F(Ab’) fragments led to significantly higher T cell transfection in vivo. However, mice receiving anti‐CD3 LNPs also had significantly reduced circulating T cell counts compared to untargeted, anti‐CD5, and anti‐CD7 LNPs.^[^ ^45^ ^]^ This phenomenon of T cell depletion by anti‐CD3 targeted LNPs has been reported by other groups and is a serious adverse effect that may hinder the effective translation of such NP platforms.^[^ ^82^ ^]^ Monoclonal antibodies like anti‐CD3 have often been used to deplete T cells, so while this targeting moiety has led to high transfection efficiency in vivo, care should be taken to use clones that are not depleting.^[^ ^83^, ^84^ ^]^ Another concern with using antibody‐decorated NPs is the potential for unwanted inflammatory side effects. Based on the stability of the NP and the NP‐antibody linkage in vivo, there is the potential the targeting antibody may dissociate from the NP before the particle has reached the immune cell type of interest.^[^ ^36^ ^]^ Not only would this prevent the desired benefit of active targeting from taking place, but the dissociated targeting moiety may then elicit unwanted responses by interacting with/depleting its target cell type or activating macrophages or NK cells that possess Fcγ receptors.^[^ ^48^ ^]^

On a broader scale, conjugating a targeting ligand to a NP, be it a PNP or LNP, requires additional processing steps and considerations that are not necessary with untargeted particles. Many of the excipients used to formulate LNPs are diluted and mixed in an ethanol phase.^[^ ^85^ ^]^ Additionally, many PNPs, such as PBAE NPs are formulated in acidic conditions.^[^ ^86^, ^87^ ^]^ These manufacturing conditions, while effective for formulating NPs, are potentially detrimental to any conjugated ligands, as many antibodies and peptides can be denatured or destroyed via exposure to acidic buffers or high ethanol concentration.^[^ ^88^ ^]^ To overcome these concerns, some researchers have followed a workflow whereby they formulate the NP with no targeting ligand, dialyze or use an ultrafiltration device to remove the potentially damaging buffers, add the targeting ligand, and then redialyze or filter the particles to remove excess ligand.^[^ ^45^, ^63^ ^]^ Although this has proven to be effective, this workflow is time‐consuming and can lead to variability in the final NP product. Additionally, it is frequently reported the conjugation of a targeting ligand leads to an increase in NP size compared to an untargeted version.^[^ ^46^ ^]^ While this is to be expected, additional research is needed to better elucidate the specific role that variations in targeted NP size play in mediating immune cell transfection in vivo. Moving forward, additional manufacturing controls are needed to ensure consistent ligand‐targeted quality as these platforms move toward clinical translation.

These potential drawbacks associated with ligand‐based targeting highlight the need to evaluate alternative NP targeting approaches to reach immune cells. Moreover, the modular nature of LNPs and PNPs enables researchers to develop diverse NP libraries and develop other targeting approaches that employ changes in NP chemistry, physical characteristics, payload, and administration to efficiently deliver nucleic acid cargo to immune cells. These alternative targeting are explored in the following sections of this review. Despite these concerns outlined above, ligand‐targeted NPs represent an incredibly exciting, viable method for gene delivery to immune cells in vivo.

## 3 Chemical Targeting

### 3.1 Motivation for Chemical Targeting

In addition to the decoration of the NP surface with macromolecular ligands, the chemical properties of the nanomaterial used to make these particles can be leveraged to modulate their tissue tropism and cell targeting capabilities. By altering the composition of the molecular building blocks, the different parameters of the NP chemical identity can be tuned, including global charge, apparent pKa, and hydrophobicity.^[^ ^26^ ^]^ This approach is motivated by well‐established methodologies which have been developed to construct libraries of NPs with differential chemical properties and to screen them according to their targeting effects in vivo. For LNPs, the canonical four‐component formulation offers a natural template starting from which researchers can introduce novel constituents or make modifications to existing ones. For many types of PNPs, the modular nature of the constituent polymers allows access to vast chemical space. The specific chemical properties of NPs are especially important to consider in the context of systemic delivery, as these chemical properties affect the formation of a protein corona in serum that then directs organ tropism.^[^ ^89^, ^90^ ^]^ Additionally, the chemical makeup of nanomaterial components plays a key role in NP uptake and endosomal escape.^[^ ^21^ ^]^

In this section, several chemical properties are outlined that can be tuned to improve NP lymphoid organ tropism and intracellular delivery specifically to immune cells, including surface charge (mediated by charged primary and secondary amines), apparent pKa (driven by ionizable secondary and tertiary amines), and hydrophobicity (often due to tunable alkyl groups). It is noteworthy that many of the studies outlined in this section all employed a similar general workflow whereby the researchers synthesized a chemically diverse library of NPs and then evaluated which formulations demonstrated the highest immune cell targeting and transfection, first in vitro and then in vivo.^[^ ^91^, ^92^, ^93^ ^]^ This approach of systematic screening based on differential chemical characteristics is frequently employed in small‐molecule drug development and nanomedicine and is increasingly being implemented in the context of NP development/gene delivery to immune cells.^[^ ^94^ ^]^ It should also be noted that different aspects of NP chemistry are sometimes coupled to one another and are difficult to vary independently. For example, two NPs with different surface charges may also have different global pKas. While this review discusses the contributions of individual chemical parameters based on the specific research study highlighted, it should be kept in mind that other parameters may not have been explicitly considered/controlled. In addition to reviewing the general strategies employed to modulate the chemical properties of NPs, recent research is highlighted that has implicated the protein corona as the potential mechanism by which chemical targeting achieves organ‐ or immune‐cell specificity.

### 3.2 Approaches

#### 3.2.1 Surface Charge

Surface charge plays an important role in mediating NP biodistribution and cell type‐specific transfection, as some cells exhibit preferential uptake of anionic NPs, while other cells preferentially take up cationic or neutral NPs.^[^ ^95^ ^]^ Several researchers have devised a variety of methods to modulate the surface charge of particles. For example, Nabar et al. coated LNPs with different polyanions via layer‐by‐layer electrostatic adsorption, imbuing the previously neutral particles with a negative charge.^[^ ^93^ ^]^ Another study by Mukalel et al. explored oxidation as a strategy to alter the LNP surface charge, whereby the researchers achieved a more positive net charge by increasing the oxidation state of the ionizable lipid.^[^ ^96^ ^]^ Different noncanonical helper lipids can also be employed to modulate NP surface charge. LoPresti et al. employed alternative cationic, anionic, or neutral helper lipids instead of the classical DOPE helper lipid when formulating LNPs, leading to the generation of a NP library with diverse surface charges.^[^ ^97^ ^]^

Emerging research suggests that introducing positive and negative surface charge to LNPs could shift the organ tropism away from the liver and to the lung or the spleen, respectively. For transfecting immune cells, spleen tropism is desirable as the spleen, being a large secondary lymphoid organ, is home to many T cells, B cells, and macrophages. Kranz et al. showed that decreasing the cationic content of RNA lipoplexes skewed biodistribution to the spleen.^[^ ^98^ ^]^ This finding was further reinforced by LoPresti et al. who observed enhanced spleen tropism in LNPs incorporating anionic helper lipids and reported a moderate negative correlation between the in vivo spleen specificity of LNPs and zeta potential.^[^ ^97^ ^]^ A critical breakthrough in chemical targeting was the invention of selective organ targeting (SORT) LNPs by Daniel Siegwart's group at the UT Southwestern Medical Center. SORT LNPs contain the four canonical lipids found in most LNPs but contain a fifth “SORT” lipid with varying charge. In a key study from Siegwart's group, Cheng et al. discovered that LNPs that incorporated a permanently cationic SORT lipid demonstrated enhanced lung tropism, LNPs with an ionizable cationic lipid demonstrated enhanced liver tropism, and LNPs with a permanently anionic lipid demonstrated enhance spleen tropism.^[^ ^99^ ^]^ These LNPs were capable of robust mRNA delivery and CRISPR‐Cas9 gene editing within the different organs in which they trafficked. Interestingly, the researchers discovered that the observed organ tropism was not specific to the exact chemical structure of the SORT lipid but was due to the overall charge of the SORT lipid used. Ultimately, the development of SORT LNPs is an excellent example of employing a screening‐based approach to understand relationships between structure and function, as the researchers synthesized a library of LNPs with different charged lipids/chemical makeups and then delivered that library in vivo to understand how these different chemistries affected biodistribution.

In terms of cellular uptake, it is established that NP charge plays a key role in mediating endocytosis.^[^ ^95^ ^]^ A study by Lunov et al. reported that phagocytic cells preferentially uptake particles presenting a negative charge, with macrophages generally demonstrating higher phagocytosis than monocytes.^[^ ^100^ ^]^ In addition, it was observed that distinct cellular pathways were activated in macrophages and monocytes. Phagocytosis mediated by CD64, an Fcγ receptor, dominated particle internalization in macrophages in a serum‐dependent manner, while dynamin II‐dependent endocytosis and micropinocytosis were the main internalization pathways in monocytes.

NP charge also affects downstream events after cellular uptake, including cargo release and cytotoxicity. Many nanocarriers make use of cationic building blocks due to their ability to efficiently complex with and encapsulate anionic nucleic acid cargoes. However, this interaction should ideally be disrupted after the nanocarrier enters the target cell to release the cargo. Additionally, polycations have been shown to be cytotoxic in various cell types upon NP uptake.^[^ ^101^ ^]^ McKinlay et al. outlined a potential solution to these concerns through the development of lipophilic charge‐altering release transporter (CART) PNPs.^[^ ^102^ ^]^ The cationic CART polymer, an oligo(α‐amino ester), self‐assembles with anionic mRNA to form a NP, but upon intracellular delivery, gets rearranged into two neutral small molecules that are less toxic to the cell. By developing a library of 64 CART NPs, the researchers were able to discover a lipophilic CART formulation that demonstrated robust T cell transfection in vivo without the need for targeting ligands. Building on this work, Li et al. developed a new library of 24 CART NPs and discovered that CART NPs with a beta‐amido carbonate backbone and altered spacing of lipophilic side chains demonstrated 97% spleen tropism when delivered systemically. Additionally, these optimized NPs were capable of improved T cell transfection in vivo.^[^ ^103^ ^]^ Like SORT LNPs, CART PNPs were discovered from a screening‐based approach where small variations in nanomaterial structure were found to impact organ tropism and T cell transfection in vivo. Moreover, by expanding on structure–function relationships discovered by McKinlay et al. Li et al. were able to design an optimized chemical formulation that demonstrated improved T cell transfection in vivo. Library‐based approaches are key to better understanding how differential chemical structures of nanocarriers generate specific biological activities, including novel chemical targeting mechanisms.

#### 3.2.2 Apparent pK_*a*_

The apparent pK_a_ of a NP has been shown to exert a multifaceted influence on its transfection efficiency. For any defined system, the experimentally measured apparent pKa is the pH at which the equilibrium concentration of protonated and unprotonated groups are equal. For a NP composed of many biomolecules and ionizable groups, the apparent pKa is an average measurement that takes into account the contributions from all of the ionizable groups within the particle. Hence, it should be noted that the intrinsic pKa value of any constituent molecule, even the predominant component of NP surface coating, may not reflect the accurate pKa of the particle itself.^[^ ^93^ ^]^ The apparent pKa controls the charged state of the NP in the serum. Apparent pKa also influences the behavior of the buffering effect the particle exerts inside the endosome once it is internalized by the target cell. It has been suggested that different cells may have different endosomal pHs.^[^ ^104^ ^]^ As such, NPs with different apparent pKas may demonstrate variable efficacy across cell types. A recent review conducted by Patel et al. provides an excellent overview of the overall effect that apparent pKa has on the efficacy and toxicity in a more general context.^[^ ^21^ ^]^ The endosomal pH generally decreases from 7.0 to 5.5 during maturation. As such, NPs with an apparent pKa within this range would exhibit a “proton sponge effect” due to the buffering effect caused by endosomal acidification/protonation, leading ultimately to endosomal escape.^[^ ^105^ ^]^ The study by Mukalel et al. using oxidized LNPs suggested the existence of an optimal range of apparent pKa between 6.5 and 7.0 for transfecting macrophages and monocytes.^[^ ^96^ ^]^

Researchers have employed different techniques to alter the apparent pKa of NPs. Nabar et al. obtained LNPs with differential apparent pKas ranging from 4.4 to 6.5 by coating the particles with different polyanions.^[^ ^93^ ^]^ While this polyanion coating process was an effective way of modulating NP pKa, this also varies NP surface charge, showing how specifically modulating one nanomaterial chemical parameter can also affect others. In a study by Mukalel et al. oxidation of the ionizable lipid was also reported as a general strategy to increase the apparent pKa of LNPs.^[^ ^96^ ^]^ Dilliard et al. manipulated the apparent pKa of LNPs by adding different SORT lipids to the canonical formulation, allowing the researchers to generate NPs with a wide range of pKas between 3.73 and >11.^[^ ^34^ ^]^

**Figure 3:**
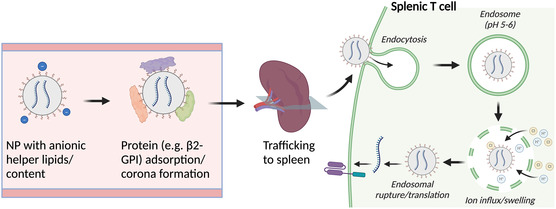
Overview of chemical targeting approaches employed for NP splenic tropism and T cell transfection in vivo. NPs with anionic surface charge have generally been found to preferentially traffic to the spleen, potentially due to the formation of a protein corona containing serum proteins like β2‐glycoprotein I (β2‐GPI). Additionally, NPs with an apparent pKa between 5 and 6 have been demonstrated to show enhanced endosomal escape and transfection in T cells. These findings can be leveraged to specifically deliver nucleic acid cargo to splenic T cells. Figure made with BioRender.

Many researchers have leveraged this ability to adjust the apparent pKa of a NP to effectively tune biodistribution and enhance splenic delivery. Nabar et al. reported that polyanion‐coated LNPs with an apparent pKa of 5.0 demonstrated improved splenic transfection compared to NPs with a higher pKa.^[^ ^93^ ^]^ In a similar study from LoPresti et al. researchers found that LNPs that incorporated a charged helper lipid with an intrinsic pKa of 5.5 demonstrated the highest transfection in the spleen, although the global pKa of the entire NP was not characterized.^[^ ^97^ ^]^ In the context of SORT LNPs, Dilliard et al. observed that the LNPs that incorporated an anionic lipid‐like 18:PA exhibited an apparent pKa between 2 and 6 and had selective delivery to the spleen.^[^ ^34^, ^99^ ^]^ Given these independent findings, it is likely that the optimal range of NP pKas for delivery to the lymphoid‐rich spleen is between 5 and 6. Building on this, Álvarez‐Benedicto specifically optimized spleen SORT LNPs for T cell engineering applications and found that LNPs with an apparent pKa of 5.88 demonstrated the highest splenic T cell transfection in vivo.^[^ ^91^ ^]^ The optimized T cell SORT LNPs were employed to generate CAR T cells in situ that promoted the overall survival of mice with B cell lymphoma. The approach employed in the development of spleen‐SORT LNPs and their use for in vivo CAR T cell engineering can be seen in **Figure** **3**. These results show that not only can NP chemical properties like apparent pKa be tuned to control biodistribution, but also this optimal biodistribution can then be leveraged to specifically transfect desired immune cell subsets.

#### 3.2.3 Hydrophobicity

In addition to being an important parameter in driving NP uptake and transfection in general, hydrophobicity is an important parameter in mediating NP recognition by the immune system in vivo. Different methodologies have been developed to adjust the hydrophobicity of PNPs and LNPs. Due to the modular nature of polymers, their hydrophobicity can be tuned by adjusting the quantity/chemical identity of different hydrophobic monomers in the nanomaterial structure. For example, Shima et al. modified the overall hydrophobicity of poly(γ‐glutamic acid) NPs by varying the grafting densities of a lipophilic sidechain on the backbone monomer structure.^[^ ^106^ ^]^ Similarly, Ben‐Akiva et al. varied NP hydrophobicity by synthesizing a series of PBAE polymers with the same backbone structure but different lipophilic side chain lengths.^[^ ^92^ ^]^ Conversely, the nonpolar nature of many lipids enables modulation of the hydrophobic properties of LNPs. This was demonstrated by Yan et al. who varied the overall hydrophobicity of LNPs by incorporating ionizable lipids with branched hydrophobic tails.^[^ ^107^, ^108^ ^]^

**Figure 4:**
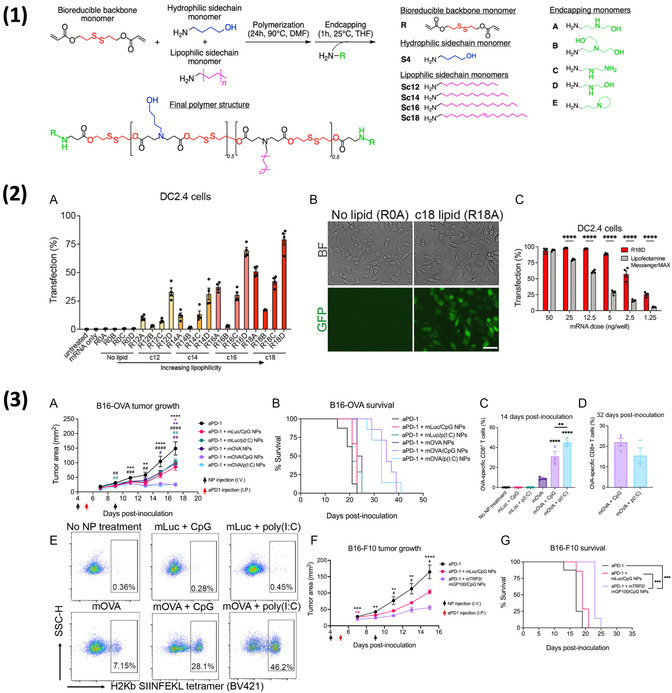
1) Overview of combinatorial PBAE library synthesis whereby a linear backbone monomer is reacted with lipophilic side‐chain monomers of variable lengths and different end‐capping monomers. 2) (A,B) Transfection efficiency of different lipophilic PBAEs was screened in DCs in vitro, with NPs possessing longer lipophilic side chains demonstrating generally higher transfection. (C) The top‐performing PBAE formulation, R18D, was also compared to Lipofectamine, where it demonstrated higher efficacy across many doses. 3) (A) R18D PBAE NPs delivering OVA‐encoding mRNA were used for in vivo cancer vaccination in a B16‐OVA melanoma model, where they demonstrated decreased tumor burden, (B) increased survival, and (C–E) robust antigen‐specific T cell expansion. (F,G) R18D NPs demonstrated similar efficacy in a cancer vaccination approach with a B16‐F10 melanoma model. Reproduced (Adapted) with permission.^[^ ^92^ ^]^ Copyright 2023, Proceedings of the National Academy of Sciences.

By varying these chemical parameters, researchers discovered that the hydrophobic properties of a particle greatly influence dendritic cell (DC) transfection in vitro and in vivo. By incorporating lipids with branched hydrophobic tails, Yan et al. developed LNPs that had a lower internal hydrophobicity and a less charged, more hydrophobic surface.^[^ ^107^ ^]^ These LNPs demonstrated enhanced DC transfection in vitro and were able to effectively elicit an anticancer response in vivo when used as a cancer vaccine. Similarly, Ben‐Akiva et al. reported that an increase in the length of the lipophilic side chain in PBAE NPs led to increased DC uptake and transfection in vitro.^[^ ^92^ ^]^ When the optimal lipophilic NPs were then delivered with antigen‐encoding mRNA and an adjuvant, they were able to successfully transfect DCs in vivo and elicit an antigen‐specific anticancer response. The approach, whereby a library of NPs with varied structures was screened in vitro, followed by an evaluation of top‐performing polymers in an in vivo therapeutic model can be seen in **Figure** **4**. Additionally, although not specifically for gene delivery applications, Shima et al. discovered that polymeric NPs with increased hydrophobicity that encapsulated antigen peptides demonstrated improved delivery to DCs and antigen‐specific immune response.^[^ ^106^ ^]^ Ultimately, these results highlight the importance of NP hydrophobicity in specifically transfecting DCs in vivo. Although the exact mechanism driving this finding is not known, DCs specifically possess innate pattern recognition receptors that may recognize the hydrophobic molecular structures in different NPs.^[^ ^109^ ^]^

#### 3.2.4 Other Chemical Parameters

Other molecular‐based targeting strategies have also been employed for gene delivery to immune cells. Elawakil et al. achieved selective targeting to splenic APCs, including B cells, macrophages, and DCs, by introducing glycidylamine‐derived lipids to the LNP formulation and optimizing their stereochemistry.^[^ ^110^ ^]^ Ni et al. inspired by the prevalence of piperazine as a motif in biologically active small molecules, synthesized a library of piperazine‐derived lipids.^[^ ^111^ ^]^ They screened the LNPs containing these lipids for transfection of splenic immune cells and were particularly successful with achieving macrophages and DCs transfection. Billingsley et al. synthesized a library of chemically distinct ionizable lipids and LNPs formulated by these lipids were screened and top performers were shown to effectively transfect primary human T cells in vitro.^[^ ^112^ ^]^ A study by Fenton et al. described the synthesis of a novel ionizable lipid dubbed OF‐deg‐Lin. LNPs formulated with OF‐deg‐Lin were shown to facilitate RNA transfection of splenic B‐cells, a cell population that has largely been unexplored for chemical targeting.^[^ ^113^ ^]^

Due to the vastness of accessible chemical space, most of the described studies employed a variation of either high‐throughput or parallel screening. Further exploration of relationships between NP chemistry and cellular targeting and uptake in immune cells would likely yield further insights and is a worthwhile direction of ongoing research.

### 3.3 Endogenous Targeting and the Protein Corona

Recent attempts to elucidate the underlying mechanisms of chemical targeting have generated accumulating evidence suggesting that the targeting outcomes of chemical modifications of NPs were in fact mediated by the protein corona.^[^ ^26^ ^]^ Such observations have motivated the adoption of a new paradigm, “endogenous targeting,” which seeks to modulate the protein corona to achieve improved targeting, often via chemical modifications to the particle.

The consideration of the protein corona has long been included in nanocarrier design. It was traditionally considered that the impact of the protein corona was undesirable, and the paradigm was to eliminate the corona completely by incorporating PEG into the NP formulation. In addition to regulating the surface charge, PEGylation also creates an antifouling hydration layer that inhibits protein adsorption on the particle surface. The improvement of delivery performance due to PEGylation was largely attributed to the reduction in the amount of adsorbed serum opsonin and the consequent downregulation of immune clearance. Nonetheless, it has been increasingly apparent that PEGylation cannot completely prevent protein corona formation, and that the effect of the corona is not entirely detrimental.

It has been shown that the ability of SORT to direct spleen tropism originated from the elevated level of β2‐GPI in the corona of the particle.^[^ ^34^ ^]^ Such an observation was supported by earlier studies showing increased uptake by macrophages.^[^ ^114^ ^]^ It was hypothesized that the recruitment of β2‐GPI can be mediated by the inclusion of anionic lipid in the formulation. Successful delivery to the immune cell population was also shown to be associated with a low content of apolipoprotein E (ApoE) in the protein corona. It was rationalized that ApoE leads to the capture of the particle by the liver through the receptor‐dependent pathway, conferring a predominant hepatic tropism. Thus, surface chemistry that resists the adsorption of ApoE might prove a viable strategy for improving immune cell transfection in in vivo.

Other indirect evidence supports the importance of protein corona as a major contributor to NP tropism. For example, the discrepancy between in vivo and in vitro studies can partly be explained by differences in or absence of the protein corona. More specifically, certain effects of chemical targeting are serum‐dependent. For instance, macrophages were shown to exhibit differential preference of uptake for surface charge with or without serum protein in the culture media, strongly suggesting that the protein corona was responsible for the selective uptake behavior.^[^ ^100^ ^]^ All listed evidence points to the protein corona as a critical link between the modification of particle surface chemistry and its biological targeting ability. Dedicated research and improved understanding of the crosstalk between particle chemical identity, protein corona species identity, and the biodistribution profile of NPs offers insight into novel avenues to further control the in vivo fate of nanocarriers.

## 4 Physical/Morphological Targeting

### 4.1 Motivation for Physical Targeting

The bulk of research focused on targeting DNA or mRNA NPs to immune cells has been focused on conjugating a synthetic ligand to the particle surface (active targeting) or modulating particle chemistry to promote interaction with endogenous proteins that alter biodistribution and delivery (passive targeting).^[^ ^26^ ^]^ While effective, these approaches come with potential translational drawbacks inherent to using macromolecular ligands or serum‐dependent biomaterials, as outlined above. Physical targeting is predicated on the notion that different immune cells will preferentially interact with different biomaterials based on their physical and morphological parameters, such as size, shape, stiffness, surface topology, etc.^[^ ^115^ ^]^ This approach is especially appealing in the context of NP‐mediated gene delivery, as the modular nature of LNPs and PNPs means that different biomaterial components can potentially be varied to preferentially transfect specific immune cell types in vivo. While there has been an in‐depth characterization of the role that these physical parameters play in mediating biomaterial interactions with immune cells, much of this work has specifically been focused on NP engineering for drug delivery and immunomodulation purposes.^[^ ^49^ ^]^ However, these fundamental discoveries regarding the structure–function relationships between nanomaterial parameters and immune cell targeting can be leveraged to engineer optimal LNPs and PNPs for in vivo immune cell gene delivery. The key physical parameters that mediate immune cell NP delivery and the strategies employed to vary these characteristics are outlined in this section.

### 4.2 Approaches

#### 4.2.1 Size

NP size has been demonstrated to play a significant role in uptake by immune cells, with differentially size particles exhibiting altered biodistribution and cellular interaction properties.^[^ ^116^, ^117^ ^]^ A model developed by Zhang et al. proposed that cells exhibit size‐dependent endocytosis, with the optimal NP diameter for efficient cellular uptake being in the 50–60 nm range.^[^ ^118^ ^]^ This is based on the concept that NPs in this size range will interact with endocytic receptors and promote optimal membrane wrapping and internalization. The model posits that NPs smaller than this range will interact with endocytic receptors but will be too small to promote membrane wrapping, while NPs larger than this will efficiently bind to the target cell, but their large size will increase cell membrane tension and sterically inhibit additional NPs from binding to the cell. While other studies have also found this ≈60 nm NP radius to be efficient for maximizing particle uptake, researchers have predominantly studied intracellular delivery in vitro in immortalized cell lines, such as HeLa cells.^[^ ^119^, ^120^, ^121^ ^]^ Many immune cells, such as macrophages, APCs, and T cells, have other endocytosis mechanisms that are preferential to NPs larger than this ≈60 nm size.^[^ ^122^ ^]^ An excellent review from Charpentier and King outlines caveolae‐dependent endocytosis, clathrin‐dependent endocytosis, and macropinocytosis as being three of the primary uptake mechanisms employed by T cells, and many immune cells in general.^[^ ^47^ ^]^ These uptake mechanisms exhibit a general size dependency, with caveolae‐mediated endocytosis employing smaller vesicles (50–80 nm), clathrin‐mediated endocytosis employing intermediate vesicles (35–200 nm), and macropinocytosis employing larger vesicles (200 nm+).^[^ ^123^, ^124^, ^125^, ^126^ ^]^

As previously discussed, macrophages are an appealing cell type for in vivo transfection due to their active endocytosis mechanisms and ability to exhibit both inflammatory and anti‐inflammatory effects.^[^ ^54^ ^]^ Although there is no definitive size range, researchers have found that particles larger than 60 nm in diameter are more effective in being taken up by macrophages in vitro and in vivo.^[^ ^127^ ^]^ A study by Petithory et al. demonstrated that larger particles in the 300–450 nm range were optimal for macrophage internalization and resulted in all particles being taken up in an in vitro coculture experiment, while only some of the smaller particles (50–200 nm) were taken up by the macrophages.^[^ ^128^ ^]^ These differences in NP internalization were attributed to the fact that macrophages exhibit different uptake mechanisms depending on particle size, with the larger 300–450 nm particles being internalized via phagocytosis, while the smaller 50–200 nm particles were internalized via clathrin or caveolae‐mediated endocytosis.^[^ ^129^, ^130^, ^131^ ^]^ Other studies have also implicated 200 nm as the cutoff size whereby monocytes, namely, macrophages, begin to internalize their targets via phagocytosis instead of endocytosis.^[^ ^127^, ^132^ ^]^ Importantly, reports also demonstrate that macrophage phagocytosis occurs on the larger 1–3 μm scale, as this is the size range of most bacteria.^[^ ^133^ ^]^ While it is unclear if NPs that large would be capable of efficient intracellular gene delivery to macrophages in vivo, these studies underscore the fact that macrophages have the potential to phagocytose larger particles (≈450 nm) that might be too large to be taken up by other cell types.^[^ ^134^ ^]^ This size bias could be leveraged to use larger DNA/mRNA NPs that may preferentially transfect macrophages in vivo.

There have been recent efforts to control the size of nucleic acid NPs to specifically uncover the relationship between particle size and efficacy. One approach by Hu et al. employed a kinetically controlled nanocomplexation process to fabricate PEI‐DNA NPs with different, controlled sizes.^[^ ^135^ ^]^ Moreover, by using this assembly process, the researchers found that differentially sized NPs demonstrated different biodistribution and transfection efficacy in an in vivo lung metastasis model. This platform for controlling nucleic acid NP size could be utilized to potentially target and transfect macrophages in the future. Moreover, this approach can be utilized to further uncover structure–function relationships between particle size and in vivo immune cell transfection.

**Figure 5:**
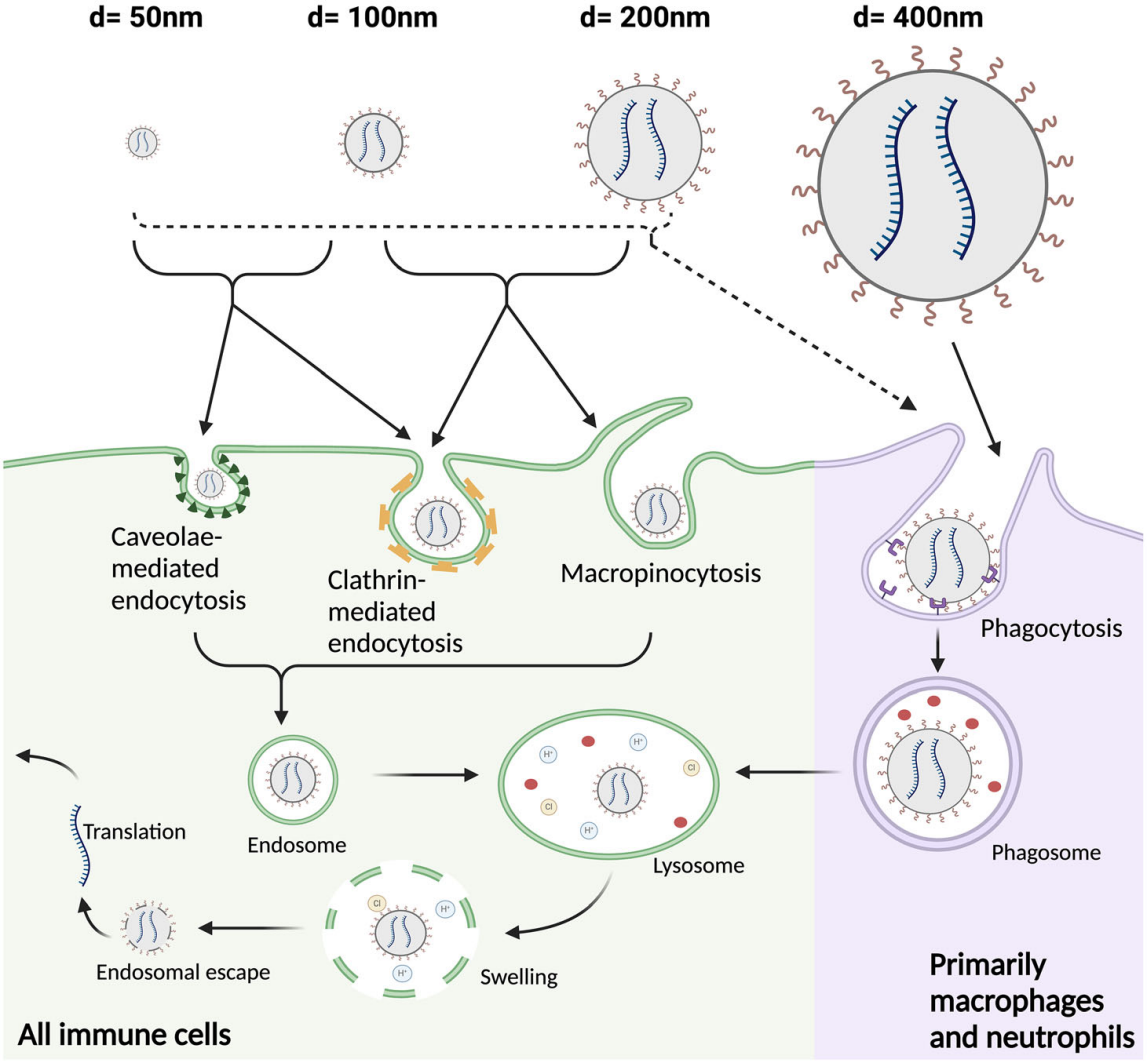
NP size influences the uptake mechanism by different immune cells. Almost all immune cells (and most cells in general) are capable of caveolae‐mediated endocytosis, clathrin‐mediated endocytosis, and macropinocytosis. Although there are exceptions, NPs in the 50–100 nm range are generally taken up via caveolae‐ or clathrin‐mediated endocytosis and may also be taken up by phagocytosis. NPs in the 100–200 nm range are primarily taken up by clathrin‐mediated endocytosis or macropinocytosis and may also be taken up by phagocytosis. Macrophages and neutrophils are capable of endocytosing larger particles (≈400 nm) via phagocytosis. Upon cellular uptake, all particles must escape endosomes in order for their nucleic acid cargo to be translated.^[^ ^47^, ^126^ ^]^ Importantly, NP uptake is a complex process, with other parameters besides size, such as charge and targeting ligand, playing a key role in determining if the immune cell employs phagocytosis, macropinocytosis, or endocytosis. Figure made with BioRender.

Unlike macrophages, most T cells do not have robust phagocytosis mechanisms, making them harder to transfect using size‐based targeting alone.^[^ ^47^ ^]^ However, pathways like macropinocytosis and clathrin‐mediated endocytosis are employed by activated T cells, most likely due to the need for large amounts of glucose and amino acid uptake upon activation.^[^ ^136^, ^137^ ^]^ Interestingly, many of the NP formulations described in the previous sections on ligand‐based targeting and chemical targeting were all in the 100–250 nm range, with no particles being significantly smaller or larger.^[^ ^40^, ^45^ ^]^ Although these NPs also employed different active and passive targeting approaches to enhance T cell gene delivery in vivo, this also points toward ≈200 nm potentially being a suitable size range for T cell targeting in vivo. Other in vitro uptake studies have demonstrated the T cell NP uptake decreases as a function of particle size, with 200 nm being on the higher end of what can potentially be internalized.^[^ ^138^ ^]^ Larger particle size for gene delivery can also be helpful to accommodate larger nucleic acid loads per particle. Ultimately, further characterization of the relationship between NP size and T cell uptake/transfection is necessary to design optimal particles for in vivo transfection. However, despite the importance of NP size in mediating T cell uptake, modulating size alone may not be sufficient to transfect T cells in vivo. Many cells in the body exhibit macropinocytosis mechanisms for uptake of ≈200 nm objects, and without any specific targeting mechanism, these particles alone may not be able to effectively engineer T cells.^[^ ^139^, ^140^ ^]^ An overview of the different NP uptake mechanisms employed by most immune cells, and the size ranges that these mechanisms are most often associated with can be seen in **Figure** **5**

As indicated in Figure 5, while phagocytosis has been employed as a targeting strategy for NPs larger than ≈400 nm, the literature shows that macrophages can also phagocytose smaller NPs as well (such as ≈30 nm).^[^ ^134^ ^]^ Moreover, it is often difficult to fully separate NP size from other physiochemical parameters like charge, ligand, opsonization, etc. These parameters also play a critical role in promoting NP uptake and determining if phagocytic mechanisms are employed by the immune cell. As such, it is important to also consider possible exceptions to the general particle size‐uptake trends outlined.

#### 4.2.2 Shape

In addition to size, NP shape plays an important role in driving interactions with immune cells and can be leveraged to enhance transfection. The majority of PNPs and LNPs used for nucleic acid delivery are roughly spherical in nature as these particles form through self‐assembly of the cationic polymer or lipid and the anionic DNA or mRNA and this shape minimizes surface area and energy.^[^ ^11^ ^]^ However, when looking at the broader landscape of NPs used in drug delivery and immunomodulation, research has shown that nonspherical particles can lead to differential interactions between the biomaterial and specific immune cell in vivo.^[^ ^141^ ^]^

For example, nonspherical NPs have been shown to be more effective at activating T cells in vivo. Ben‐Akiva et al. discovered that prolate ellipsoidal PLGA artificial antigen‐presenting cell (aAPC) NPs demonstrated enhanced biodistribution properties and reduced nonspecific uptake by non‐T cells, allowing these particles to better interact with T cells in vivo and lead to improved tumor reduction.^[^ ^142^ ^]^ This ellipsoidal NP shape is beneficial for targeting T cells in vivo because this geometry promotes greater interaction between the particle surface ligands and the T cell. Additional research suggests that nonspherical particles may demonstrate enhanced biodistribution and in vivo persistence due to their ability to evade the mononuclear phagocytic system (MPS).^[^ ^143^, ^144^ ^]^ Conversely, particles can also be engineered to promote phagocytosis and delivery to macrophages.^[^ ^145^ ^]^ Although macrophages have been shown to uptake particles of various shapes, Champion et al. demonstrated that the local geometry of the point of contact between the macrophage and the particles plays a critical role in if the cell will perform phagocytosis.^[^ ^146^ ^]^ Formulating nonspherical DNA or mRNA NPs may also prove to be challenging, as most PNPs and LNPs are formed via bulk electrostatic interactions between the cationic lipid/polymer and the anionic nucleic acid that promote spherical self‐assembly.

#### 4.2.3 Stiffness

Although the size and shape of a NP have generally been demonstrated to play a larger role in mediating NP interactions with different immune cells, there are other physical parameters that have also been discovered to alter NP function and delivery profile, such as particle stiffness. Like size and shape, stiffness has been investigated in the context of nanomedicine and immunoengineering as a whole, but it has had little investigation specifically in the context of nucleic acid NP intracellular delivery.^[^ ^147^ ^]^

**Figure 6:**
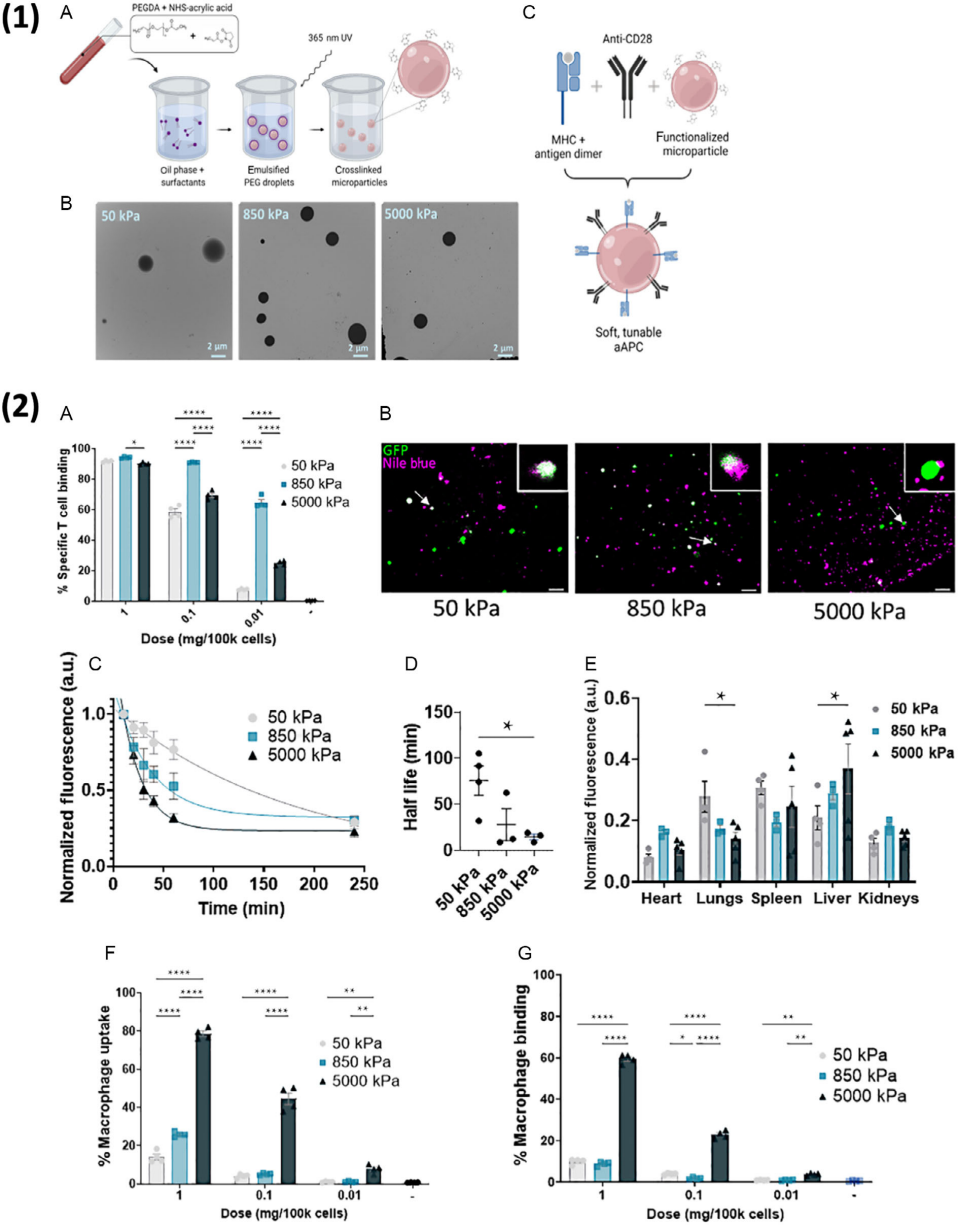
1) (A) Overview of elastic PEG aAPC synthesis whereby crosslinker amount was varied to modulate PEG microparticle stiffness. (B) Microparticles (MP) were characterized with transmission electron microscopy and (C) conjugated to T cell‐activating molecules to form aAPCs. 2) Binding and uptake of aAPCs with varying stiffnesses were evaluated in vitro. (A) The intermediate (850 kPa) MPs demonstrated higher specific T cell binding at low doses, with interactions between T cells (green) and MPs (pink) being demonstrated in microscopy images in (B). (C,D) When administered in vivo, the softest MPs (50 kPa) demonstrated the longest half‐life and (E) enhanced delivery to the lung and spleen, while the stiffest particles (5000 kPa) demonstrated enhanced delivery to the liver. (F) Additionally, the stiffest 5000 kPa MPs demonstrated the highest macrophage uptake and (G) binding in vitro. Reproduced (Adapted) with permission.^[^ ^148^ ^]^ Copyright 2024, Nano Research.

Recent research has worked to elucidate the role of particle stiffness on immune cell targeting in vivo. A study by Est‐Witte et al. discussed the role of modulating aAPC microparticle elasticity on T cell stimulation in vivo.^[^ ^148^ ^]^ The researchers studied the function of aAPCs with low (50 kPa), intermediate (850 kPa), and high (5000 kPa) stiffnesses. Softer particles (50 kPa) demonstrated improved half‐lives while intermediate particles (850 kPa) demonstrated higher‐specific T cell binding and lower macrophage binding/uptake in vitro. Additionally, these intermediate particles induced the highest antigen‐specific T cell expansion in vivo. Interestingly, particles with different stiffnesses demonstrated different protein corona profiles in serum, suggesting that this biomaterial physical property affects serum interactions in vivo, which in turn can affect biodistribution and efficacy. While this study was not specifically performed in a gene delivery context, these findings regarding the effect of biomaterial stiffness on biodistribution, uptake/binding, and T cell activation in vivo can easily be applied to mRNA/DNA NPs. An overview of this experimental methodology, whereby polymeric particles with different stiffnesses were synthesized and then evaluated for their immune cell binding ability in vitro can be seen in **Figure** **6**. In a similar study, Kong et al. investigated the role of varying layer‐by‐layer (LbL) liposomal NP stiffness on biodistribution.^[^ ^149^ ^]^ The researchers found that compliant LbL NPs with an elastic modulus between 6 and 8 kPa demonstrated improved circulation time, lower filtration via the kidneys, and greater accumulation in tumors than stiff NPs with a modulus between 21 and 24 kPa. In gene delivery contexts, properties like NP stiffness can be altered by varying NP components like the cholesterol content or polymer structure.^[^ ^150^ ^]^ As such, these insights regarding the in vivo effect of aAPC and LbL NP stiffness can be easily translated to LNPs and PNPs.

Certain physical properties have also been shown to help NPs evade uptake by macrophages, which could be beneficial when trying to target these particles to other immune cells, like T cells.^[^ ^147^, ^151^ ^]^ Zou et al. demonstrated that softer NPs demonstrate lower uptake by macrophages, thereby enhancing circulation time and tumor penetration.^[^ ^152^ ^]^ This was supported by Guo et al. who observed favorable therapeutic outcomes with softer lipid‐based NPs due to the fact that they preferentially accumulated in the tumor site, while elastic/harder NPs trafficked to the liver.^[^ ^153^ ^]^ Additionally, softer NPs, being more flexible in nature than hard NPs, are better suited for lymphatic delivery due to their ability to squeeze through blood and lymphatic vessels.^[^ ^154^ ^]^ These softer NPs may also be better suited for intracellular delivery, as research suggests that a softer particle is optimal for diffusion across a lipid bilayer and penetration into the cell.^[^ ^20^, ^155^ ^]^ This may be of greater importance for LNP gene delivery compared to PNP delivery, as the lipid‐based nature of an LNP enables the particles to fuse with and integrate into the cell membrane to enhance intracellular nucleic acid delivery.^[^ ^20^ ^]^

#### 4.2.4 Surface Morphology

NP surface topology also mediates circulation time and biodistribution. In addition to size, interactions between NPs and cells are shown to be driven by the membrane tension and curvature of the target cell.^[^ ^156^, ^157^ ^]^ It has been shown that having positive relative curvature between the NP and cell, meaning that both the particle and cell curve to the same degree at the point of contact, is favorable for enhancing endocytosis. Many immune cells, such as T cells and NK cells are spherical in nature, while DCs and macrophages have multiple points of relatively higher curvature.^[^ ^158^, ^159^ ^]^ As such, modulating the surface curvature of NPs could help in improve delivery to specific immune cells in vivo. Additionally, modulating/masking NP surface charge is critical for improving particle half‐life and circulation in vivo. Most NPs that are delivered intravenously are coated with a charge‐shielding material that imparts a neutral charge on the overall particle.^[^ ^160^ ^]^ PEG is most often used for charge shielding applications, as it is already one of the four components that make up LNPs and can easily be incorporated into many PNPs.^[^ ^161^ ^]^ By neutralizing the surface charge of the NP, PEG promotes stability and prevents aggregation, which is an important consideration for manufacturing and quality control.^[^ ^162^ ^]^ Upon administration, PEGylation helps reduce opsonization by antibodies and complement system intermediates that are present in serum, thereby improving circulation time.^[^ ^163^ ^]^ Given the integral role that PEG plays in LNP structure, these considerations regarding surface charge shielding are already being applied in the context of in vivo gene delivery and should continue to be implemented.

## 5 Cargo‐Mediated Targeting

### 5.1 Motivation for Cargo‐Mediated Targeting

The specific nucleic acid cargo of DNA and mRNA NPs can also affect the ability of these particles to specifically engineer function in immune cells in vivo. The discovery of nucleoside‐modified mRNA has led to groundbreaking achievements in mRNA delivery, highlighted by the development of mRNA vaccines against COVID‐19 and the Nobel Prize in Medicine being awarded to Drew Weissman and Katalin Karikó in 2023.^[^ ^164^, ^165^ ^]^ Although the more “conventional” approaches covered in the ligand, chemical, and physical‐based targeting sections are effective methods for targeting and engineering immune cells, these targeting approaches are not perfect and can still result in off‐target gene delivery in vivo.^[^ ^166^ ^]^ Cargo‐mediated targeting can potentially address these concerns by engineering the actual nucleic acid inside the particle.^[^ ^167^ ^]^ By altering the sequence of the therapeutic mRNA or DNA or even the nucleic acid structure itself, LNPs and PNPs can be designed to preferentially engineer certain immune cells and minimize off‐target effects.

### 5.2 Approaches

#### 5.2.1 Cell Type‐Specific Promoters

The risk of off‐target gene delivery is especially concerning in the context of CRISPR‐Cas9 delivery or DNA delivery with a transposon, as unwanted gene integration can potentially be harmful.^[^ ^168^, ^169^ ^]^ One method for reducing off‐target gene expression and improving the specificity of NP engineering is by incorporating a cell type‐specific promoter into the nucleic acid construct. A promoter is found before the region of the nucleic acid encoding for the therapeutic construct and is critical for enabling the expression of the therapeutic region of the gene.^[^ ^170^ ^]^ Additionally, there are many cell‐type specific promoters that enable expression of the gene construct only in specific cells, meaning that there will be no expression if the gene is delivered to an off‐target cell. This concept has already been applied for selective LNP‐mediated delivery in ocular gene therapies through the delivery of a therapeutic plasmid flanked by a retina‐specific promoter.^[^ ^171^ ^]^ This concept of selective gene transcription can easily be applied to many immunotherapies where the expression of the therapeutic construct can be altered by the presence of a specific promoter.^[^ ^172^ ^]^

**Figure 7:**
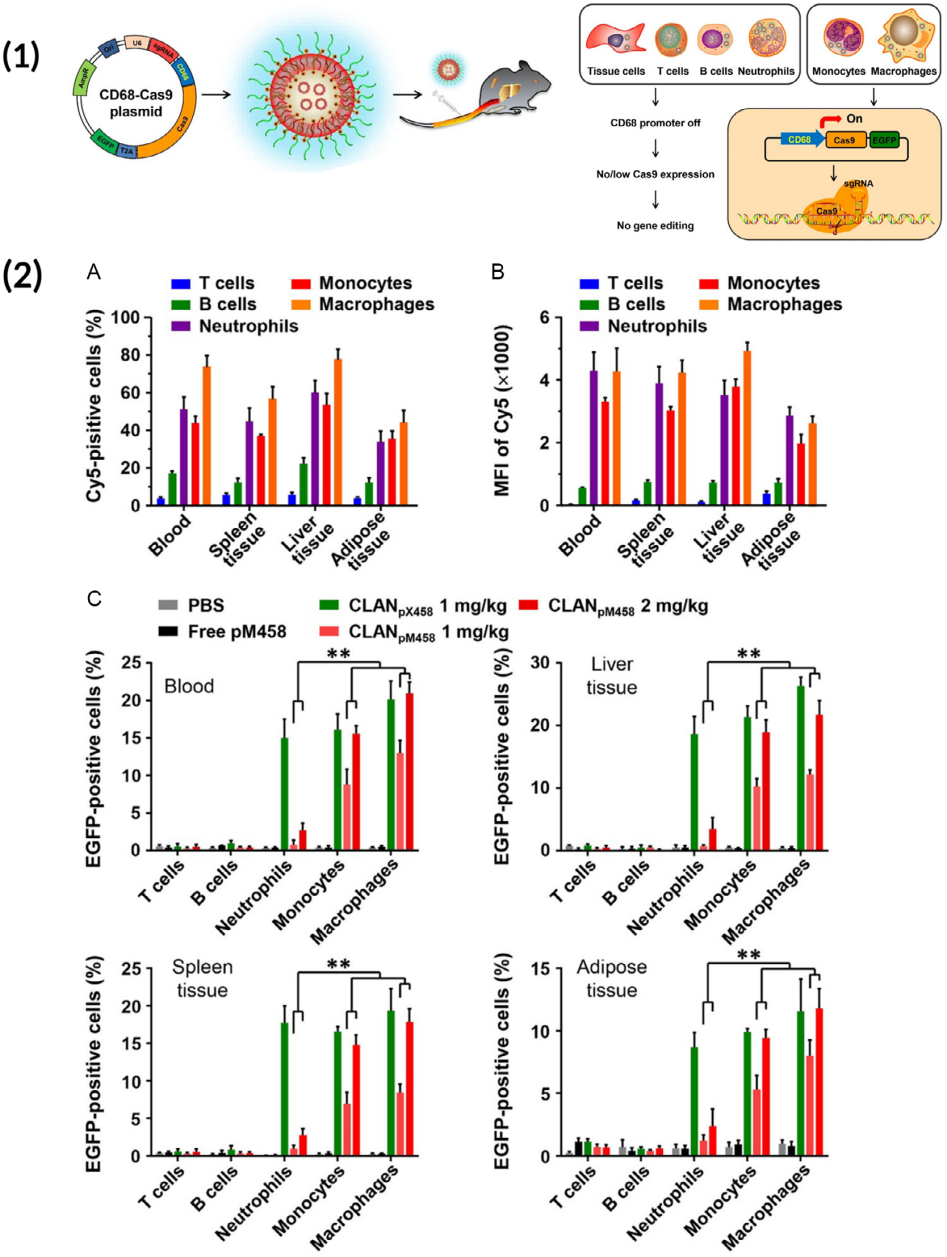
1) Overview of PNP with CD68‐promoter pDNA design whereby CRISPR‐Cas9 enhanced green flourescent protein (EGFP) plasmid will only be translated in macrophages and monocytes, even if NPs are taken up by other cell types. 2) (A,B) PNPs delivering Cy5‐labeled nucleic acid were delivered systemically, where they demonstrated robust uptake in neutrophils, macrophages, and monocytes across different organs. (C) However, only NPs with the pM458 plasmid containing the CD68 promoter demonstrated robust EGFP transfection in macrophages and monocytes in vivo, while NPs with the control pX458 plasmid also transfected neutrophils. Reproduced (Adapted) with permission.^[^ ^211^ ^]^ Copyright 2018, ACS Nano.

For example, Luo et al. developed polymeric NPs that contained a CRISPR‐Cas9 encoding plasmid with a CD68 promoter that was specific to macrophages and monocytes.^[^ ^173^ ^]^ Although these NPs were taken up by many different cells upon intravenous administration, only monocytes and macrophages, cells that express CD68, were able to turn on the promoter and express the plasmid construct.^[^ ^58^ ^]^ This cell type‐specific CRISPR expression was then leveraged to specifically disrupt the expression of a gene associated with macrophage response in type‐II diabetes, the Ntn1 gene, and reduce disease burden. This concept of genetic cargo only being translated in specific target cells is a key safety consideration. Beyond being involved in macrophage‐mediated effects of type‐II diabetes, Ntn1 has been shown to be important in regulating angiogenesis and vasculature function, suggesting that the deletion of the Ntn1 gene in other cells could be detrimental.^[^ ^174^ ^]^ However, the use of a macrophage‐/monocyte‐specific promoter in this study prevented off‐target Ntn1 deletion. The overall approach employed by the researchers in this study to engineer NP cargo to enable macrophage‐specific gene editing can be seen in **Figure** **7**.

#### 5.2.2 In Vitro‐Transcribed (IVT) *mRNA*

Synthetic mRNA is inherently inflammatory and provides several challenges for stable transfection and therapeutic protein expression in cells.^[^ ^175^, ^176^ ^]^ The development of IVT mRNA addressed these key delivery challenges and enabled noninflammatory gene delivery by modifying components of this nucleic acid.^[^ ^177^, ^178^ ^]^ For example, by changing the structure of uridine to a synthetic nucleoside like pseudouridine (Ψ) or *N1*‐methyl‐pseudouridine (m1Ψ), the synthetic mRNA was no longer immunogenic and was able to be stably expressed in the target cell.^[^ ^179^, ^180^ ^]^ IVT mRNA is critical for delivery to immune cells and has already been utilized in many in vivo transfection studies, several of which have already been covered in previous sections of this review.^[^ ^46^, ^63^ ^]^ Given the importance of this nucleoside‐modified mRNA in enabling intracellular delivery, there is further scope for optimizing this mRNA to better engineer immune cells in vivo. A study by Ye et al. specifically investigated the effect of single‐nucleotide mRNA modifications on LNP‐mediated gene delivery to macrophages and T cells.^[^ ^181^ ^]^ The researchers developed a library of LNPs delivering different modified eGFP mRNA to immortalized macrophages through m5C, m1ψ, ψ, or 5‐moU single nucleotide modifications. Interestingly, only NPs with m1ψ‐modified mRNA demonstrated robust macrophage transfection. This top formulation was then shown to efficiently transfect primary macrophages and T cells with a therapeutic CAR construct. Ultimately, these ex vivo results highlight the importance of optimizing IVT mRNA structure for immune cell transfection. Different nucleotide modifications, such as the uridine substitutions outlined above, have the potential to modulate immunogenicity, stability, translation, and overall therapeutic efficacy.^[^ ^112^, ^182^ ^]^

## 6 Delivery Route‐Mediated Targeting

### 6.1 Motivation for Delivery Route‐Mediated Targeting

Ligand‐, chemical‐, physical‐, and cargo‐mediated targeting all generally work to optimize immune cell targeting on the cellular scale, whereby different NP parameters can be altered to enhance favorable interactions between the biomaterial and the cell that lead to immune cell‐specific transfection. Unlike these methods, delivery route‐mediated targeting functions on a higher, whole‐body scale. While not a traditional form of NP “targeting”, this concept is predicated on the idea that different routes of administration can enhance particle delivery to different immune‐rich secondary lymphoid organs. Additionally, different biomaterials, including hydrogels and biocompatible implants are being developed to aid in nonviral gene delivery to immune cells in vivo. In this section, the different advantages and disadvantages associated with several key routes of NP administration are outlined and other biomaterial‐based approaches that have been employed to enhance immune cell transfection and NP release kinetics are discussed.

### 6.2 Approaches

#### 6.2.1 Different Routes of NP Administration

NPs can be delivered systemically or locally. While both administration routes have their advantages, most LNPs and PNPs used for transfection of immune cells in vivo are administered intravenously (i.v.).^[^ ^11^, ^183^ ^]^ This systemic delivery allows transfection of immune cells in hard‐to‐reach organs distributed throughout the body that may not be accessible through local administration, such as the spleen and lymph node. Although i.v. administration can often result in NP accumulation and transfection in the liver, this systemic delivery can also lead to robust delivery in the spleen.^[^ ^98^ ^]^ Moreover, there are many obstacles associated with systemic NP delivery to organs besides the spleen, including clearance, stability, extravasation, and nonspecific uptake.^[^ ^183^ ^]^ As previously outlined throughout this article, many engineering approaches have been utilized to optimize the biodistribution of i.v. delivered NPs to organs like the spleen for immunoengineering applications. Inhaled, or intranasal (i.n.) delivery of NPs has also been ably utilized for delivery to different organs in a systemic manner, though this approach is most effective for transfecting cells in the lung.^[^ ^184^ ^]^ Although more work is needed to explore i.n. administration as a potential route for transfecting immune cells like T cells and macrophages, this approach has been utilized to deliver mRNA to other immune cells in the lung. Suberi et al. developed a poly(amine*‐*co‐ester) NP that was able to transfect APCs in the lung with mRNA encoding for the SARS‐CoV‐2 spike protein.^[^ ^185^ ^]^ This elicited a systemic mucosal immune response against the SARS‐CoV‐2 virus, demonstrating how i.n. delivery can also be a potent method for delivering DNA or mRNA NPs to specific immune cells.

Although many local administration routes, such as subcutaneous (s.c.) or intradermal (i.d.) delivery are relatively simple to perform and can result in high transfection at the injection site, they may not necessarily result in robust systemic immune cell transfection.^[^ ^186^, ^187^ ^]^ One major advantage of local administration, however, is that NPs delivered s.c. have the potential to drain to the lymph nodes before they enter circulation.^[^ ^188^ ^]^ For example, Chen et al. screened several LNPs to find a formulation that demonstrated robust transfection in the lymph nodes after s.c. injection.^[^ ^189^ ^]^ This optimal LNP demonstrated lower transfection in the liver and higher transfection in the lymph nodes, with robust transfection of DCs and macrophages in the LN. In a cancer vaccine model where NPs delivering antigen‐encoding mRNA were injected s.c., these NPs elicited an antigen‐specific CD8+ response that helped prevent or control tumor growth. While this approach did not directly compare s.c. injection to other delivery routes, it does demonstrate how s.c. delivery can lead to enhanced NP transfection in the LN, which can then be used to engineer immune cells in these lymphoid organs.

Moreover, size is a critical factor in enabling NP drainage to the lymph node, with smaller NPs between 5 and 50 nm in diameter being reported as optimal for lymph node drainage.^[^ ^190^ ^]^ This is because NPs larger than 50 nm tend to get trapped in the extracellular matrix upon s.c. delivery. However, other researchers suggest that a suitable size range for NP diffusion to lymphatics from nonfenestrated capillaries is between 10 and 100 nm^[^ ^191^ ^]^ Interestingly, the NPs employed by Chen et al. that demonstrated the highest transfection in the lymph node were ≈110 nm in diameter.^[^ ^189^ ^]^ Beyond s.c. injection, intradermal administration is also an appealing approach, as there are many immune cells, especially APCs, that reside under the skin. Physical delivery technologies, such as microneedle arrays, take advantage of this to specifically deliver NPs and nucleic acids i.d., primarily for vaccine applications.^[^ ^192^, ^193^ ^]^

#### 6.2.2 Assistive Biomaterials for NP Delivery

In addition to the NP itself, different biomaterials can be used to aid in delivering the nucleic acid to the immune target of interest.^[^ ^194^ ^]^ Hydrogels conjugated to different functional molecules have previously been utilized as platforms for T cell activation and expansion as these materials provide many of the mechanical and activating cues needed for T cell stimulation.^[^ ^195^ ^]^ Given that T cell preactivation has been shown to improve uptake and transfection, these stimulating hydrogels could be combined with nucleic acid NPs to improve delivery to T cells. A study by Agarwalla et al. outlined the development of a multifunctional alginate scaffold, MASTER, that presented T cell activating signals and encapsulated CAR‐encoding viral particles.^[^ ^196^, ^197^ ^]^ When this scaffold was mixed with naïve T cells and immediately implanted s.c., the researchers found that MASTER was able to effectively activate, transduce, expand, and release functional CAR T cells in vivo that improved antitumor survival compared to traditional i.v. CAR T administration. Although this study utilized viral particles for T cell engineering, this is an exciting example of how gene delivery vectors and activating hydrogels can be used for in vivo therapeutic T cell manufacturing.

**Figure 8:**
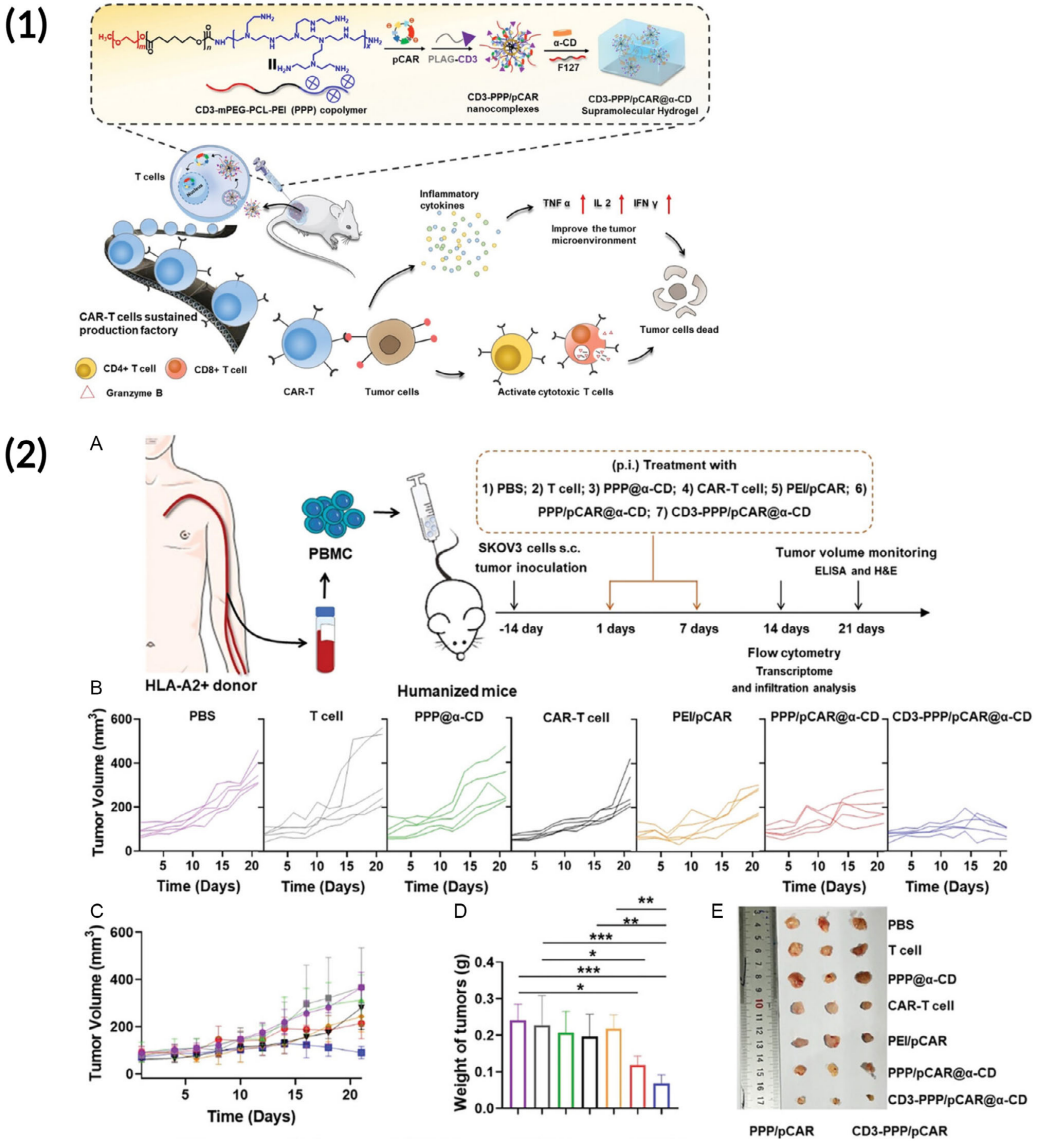
1) Overview of CD3‐mPEG‐PCL‐PEI/pCAR hydrogel design where supramolecular hydrogel could be implanted proximal to the tumor site to transfect endogenous T cells to generate antitumor CAR‐T cells. 2) (A) Humanized mice were inoculated with tumors and treated with PBS, nontransduced T cells, hydrogel with no plasmid, ex vivo generated CAR‐T cells, PEI/plasmid NPs, NP‐hydrogel with plasmid but no CD3, or NP‐hydrogel with plasmid and CD3. (B–E) The CD3‐mPEG‐PCL‐PEI/pCAR supramolecular hydrogel treatment resulted in the greatest reduction of solid tumor burden. Reproduced (Adapted) with permission.^[^ ^196^ ^]^ Copyright 2023, Advanced Materials.

A similar, nonviral approach for in situ CAR T cell manufacturing was employed by Zhu et al. whereby the researchers developed a PNP‐hydrogel composite to reprogram tumor‐infiltrating lymphocytes.^[^ ^196^ ^]^ This composite consisted of a NP that was formed via electrostatic interactions between a CAR‐encoding plasmid and a mPEG‐PCL‐PEI cationic polymer that was conjugated to anti‐CD3. This PNP was then mixed with α‐cyclodextrin and pluronic F127 to form a supramolecular hydrogel. Although this NP‐hydrogel composite was able to transfect and generate functional CAR‐T cells in vitro, when injected s.c. near the tumor site, administration of this biomaterial only resulted in modest tumor suppression. The approach employed by the authors can be seen as outlined in **Figure** **8**. Notably, this nonviral approach does not involve adoptively transferred cells like the study by Agarwalla et al. and is an exciting proof of concept regarding the use of polymeric, supramolecular biomaterials to perform the critical CAR T cell manufacturing steps with endogenous T cells in vivo.

In addition to serving as functional microenvironments for immune cell reprogramming, hydrogels have often been utilized for localized and controlled release of nucleic acid NPs.^[^ ^194^ ^]^ This is important, as off‐target NP delivery and transfection can potentially be harmful. NP release kinetics from hydrogels in vivo can be tuned by modulating the gel pore/mesh size, the degradation profile of the gel, and the relative affinity between the NP and the gel. Release kinetics can be further tuned by engineering pH, enzyme, or light‐responsive gels that function differently in in vivo settings. These kinds of nucleic acid NP‐hydrogel composites have been extensively used in the context of oncology and tissue regeneration.^[^ ^198^, ^199^, ^200^ ^]^ Recent work has sought to apply this approach for immunomodulation, with different NP‐hydrogel composites demonstrating great efficacy at attracting DCs and macrophages for vaccination and in situ CAR‐macrophage generation, respectively.^[^ ^201^, ^202^ ^]^ Although localized, hydrogel‐based delivery may not be optimal when trying to specifically target cells systemically/in the bloodstream, the use of assistive biomaterials to modulate NP release and transfection kinetics is an exciting area for future exploration.

## 7 Conclusion and Future Work

While NPs have long been used as viable platforms for gene and drug delivery in vivo, they have demonstrated poor efficacy in specifically transfecting many immune cells. Moreover, these NPs suffer from rapid clearance by the MPS, off‐target nonimmune cell transfection, and hepatic tropism in vivo. Despite these limitations, the modular nature of NP synthesis has allowed researchers to generate large NP libraries with diverse structural and chemical features that demonstrate enhanced gene delivery to immune cells. Through in‐depth analysis of structure–function relationships outlined in recent literature, four critical NP parameters that can be modulated to improve lymphoid biodistribution and on‐target immune cell transfection are: NP surface ligand, nucleic acid cargo, chemical characteristics, and physical characteristics. Additionally, NP delivery to immune cells can also be enhanced by optimizing the route by which the NPs are delivered to the body.

While the studies discussed in this review have generally evaluated these targeting strategies to improve immune cell delivery independently, future NPs should incorporate several, if not all, of the approaches outlined above to best improve specific delivery to a desired immune cell subset. For example, when designing NPs for macrophage transfection, promising particles could include the following attributes: ≈400 nm in diameter to trigger phagocytosis over endocytosis and enhance nucleic acid loading, have a targeting moiety conjugated to the surface such as dimannose that can bind to CD206 on the macrophage, and have a macrophage‐specific promoter sequence as part of the NP's DNA cargo. Alternatively, the optimal particle for T cell transfection in vivo may be smaller (100–200 nm), have an apparent pKa between 5 and 6, and be conjugated to a T cell‐specific ligand like anti‐CD3. Moreover, there is a need for future combinatorial studies that evaluate multiple targeting approaches in a single NP to assess the relative importance that each of these approaches plays in mediating transfection.

Additionally, many of the approaches for in vivo immune cell engineering outlined in this review focused primarily on T cells and macrophages. This is largely due to the fact that the overwhelming bulk of published research on gene delivery to immune cells in vivo focuses on these two cell types. While T cells and macrophages are appealing due to their roles as key effectors of adaptive and innate immunity, respectively, many other immune cell types like NK cells, B cells, and neutrophils have great therapeutic potential.^[^ ^57^, ^203^ ^]^ Extensive future characterization of different NP formulations that demonstrate robust transfection of these different cells is needed going forward.

A future critical next step for the field of nonviral gene delivery to immune cells is the integration of machine learning approaches to inform NP design. As outlined in this review, there is an increasingly large amount of data detailing structure–function relationships between different nanomaterial parameters and their ability to transfect immune cells in vitro and in vivo. These in vitro and in vivo datasets should be implemented in *in silico*/machine learning models to identify optimal NP formulations for transfecting specific immune cell types.^[^ ^204^, ^205^ ^]^ While some computational NP design approaches have recently been implemented, they have largely been focused on gene delivery to different immortalized cell lines. For example, Gong et al. implemented a large library of PBAE NPs with diverse chemical characteristics and previously measured transfection efficacies in a random forest model.^[^ ^206^ ^]^ This model was then able to identify PBAE chemical characteristics that were optimal for transfecting RAW 264.7 macrophages and Hep3B liver cancer cells. Employing a similar approach, Cheng et al. developed a machine learning model that outlined key structure–function relationships between LNP parameters and their ability to transfect six different cell types.^[^ ^207^ ^]^ This model identified that varying certain key chemical characteristics like ionizable lipid molar ratio, helper lipid molar ratio, and helper lipid chemical structure greatly affected the LNP's ability to differentially transfect a cell type of interest. These findings were then evaluated in vitro to develop NPs that demonstrated enhanced cell type‐specific transfection. Many of these recently published data‐driven approaches focus on gene delivery to immortalized cells due to their relative ease in growing up enough cells to collect large transfection datasets. However, comparably large datasets are being rapidly generated on gene delivery to primary immune cells which will enable similar computational approaches to be applied to these cells. In addition to employing machine learning to inform nanomaterial design in vitro, *in silico* modeling approaches can also be employed based on in vivo data sets detailing biodistribution on both the organ and cellular scale to further inform understanding of the key drivers of successful NP delivery to different organs and cells.^[^ ^208^, ^209^ ^]^

Finally, the development of different high‐throughput screening tools will further accelerate the speed at which different NP formulations can be generated and evaluated.^[^ ^210^ ^]^ In turn, these novel formulations can then be used to further train and optimize machine learning models for designing even better NPs. This positive feedback cycle is a key to future NP discovery and will advance this rapidly growing field of gene delivery to immune cells in vivo.

## Conflict of Interest

J.J.G. is a co‐founder, manager and CTO of Dome Therapeutics, co‐founder, board member and CSO of Cove Therapeutics, co‐founder of WyveRNA Therapeutics, and on the scientific advisory board of Mana.bio. These potential competing interests are managed by the Johns Hopkins committee on outside interests.
